# Shallow magma diversions during explosive diatreme-forming eruptions

**DOI:** 10.1038/s41467-018-03865-x

**Published:** 2018-04-13

**Authors:** Nicolas Le Corvec, James D. Muirhead, James D. L. White

**Affiliations:** 1 Laboratoire Magmas et Volcans, Université Clermont Auvergne—CNRS—IRD, OPGC, 6 Avenue Blaise Pascal, 63178 Aubière Cedex, France; 2 Department of Earth Sciences, Syracuse University, 204 Heroy Geology Laboratory Syracuse, Syracuse, NY 13244 USA; 3 Geology Department, University of Otago, PO Box 56 Dunedin, Otago, 9054 New Zealand

## Abstract

The diversion of magma is an important mechanism that may lead to the relocation of a volcanic vent. Magma diversion is known to occur during explosive volcanic eruptions generating subterranean excavation and remobilization of country and volcanic rocks. However, feedbacks between explosive crater formation and intrusion processes have not been considered previously, despite their importance for understanding evolving hazards during volcanic eruptions. Here, we apply numerical modeling to test the impacts of excavation and subsequent infilling of diatreme structures on stress states and intrusion geometries during the formation of maar–diatreme complexes. Explosive excavation and infilling of diatremes affects local stress states which inhibits magma ascent and drives lateral diversion at various depths, which are expected to promote intra-diatreme explosions, host rock mixing, and vent migration. Our models demonstrate novel mechanisms explaining the generation of saucer-shaped sills, linked with magma diversion and enhanced intra-diatreme explosive fragmentation during maar-diatreme volcanism. Similar mechanisms will occur at other volcanic vents producing crater-forming eruptions.

## Abstract (web summary)

Shallow magmatic feeder systems in monogenetic volcanic fields may determine how a volcano erupts. Here, the authors use numerical modeling to show that explosive excavation and infilling of eruptive craters affects local stress states, with feedbacks controlling sites and depths of crater-forming explosions.

## Introduction

Recent field and geophysical studies have revealed complex networks of sub-vertical dikes to sub-horizontal sills underlying monogenetic volcanic fields^1–5^, with the growth of these networks affecting the location and style of eruptive activity. Hazardous vent-site shifts are documented for monogenetic eruptions^6,7^, resulting from lateral magma diversion during growth of dike and sill feeders^3,8^. Magma diversions and transitions in intrusion geometries can be explained by several physical and structural factors, such as mechanical contrasts^9^, pre-existing fractures^10^, and stress loading/unloading^11,12^. These diversions can modulate eruptive behavior between explosive and effusive activity^13^, resulting from changes in magma-water ratios^3^, reduced internal magma pressure causing volatile exsolution^14,15^, and/or a build-up of volatiles at dike-sill junctions^1^. The combination of explosive volcanic activity (e.g., base surges)^16–18^ and potential vent-site shifts^6,8^, which gradually increase the surface region impacted by an eruption, is highly hazardous to densely populated areas situated on volcanic fields (e.g., Mexico City, Mexico, and Auckland, New Zealand). The feedbacks between magma plumbing system development and the location of magma fragmentation must therefore be well understood to forecast and mitigate volcanic hazards^19,20^.

**Fig. 1:**
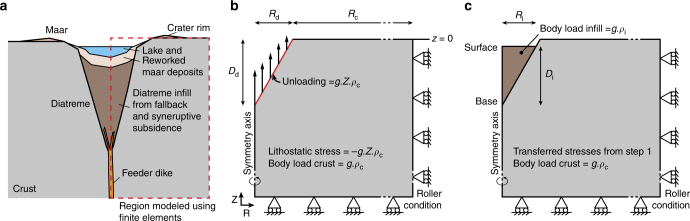
Finite element model configuration. **a** Sketch of a maar–diatreme complex. Modified from Lorenz^33^. The dotted red square represents the area modeled numerically using an axisymmetric axis. **b** The initial step models the excavation stage. The model is gravitationally loaded with a lithostatic pre-stress and a body load. A vertical load acting on the diatreme’s wall represents the mass of rock excavated. **c** The second step models the infilling stage. The initial stress conditions and geometry are transferred from the initial step. The diatreme is filled with either 25, 50, or 75% of the total diatreme volume. Physical parameters are *E*_c_ = 15 GPa, *η*_c_ = 0.25, and *ρ*_c_ = 2300 kg m^−3^ for the crust, *E*_i_ = 2.5 GPa, *η*_i_ = 0.25, and *ρ*_i_ = 2000 kg m^−3^ for the infill

The development of shallow feeder systems is increasingly recognized as playing an important role in modulating eruptive activity^3,21,22^. However, no studies to date have modeled the succession of topographic (excavation) and material (infilling) changes occurring during an explosive eruptive sequence, and its influence on the development of underlying feeder systems. Previous studies demonstrate that unloading during crustal stretching^11^, volcanic mass-wasting^23^, and caldera collapse^24^ has a profound effect on intrusion pathways. Such events, however, occur rarely or over long timescales, whereas cratering and infilling processes are common to all explosive eruptions. Here we utilize, for the first time, finite element modeling to analyze the evolution of stress states during explosive excavation and filling of gravitationally loaded country rock. Although the process of crater excavation and infilling is common to all explosive eruptions, the most strongly crater-dominated eruptions, of maar-diatreme volcanism, are chosen for this study. This allows us to test how local stress fields, and therefore magma propagation, respond to the mechanical changes produced by excavation of maar-diatreme structures that are common in mafic and kimberlite volcanic fields (Fig. 1a). We conclude that stress fields generated from maar-diatreme volcanism can cause magma diversions, with diverted magma producing intrusions with a variety of geometries, which will affect the location of magma fragmentation sites and surface eruptions.

## Results

### Stress states affecting intrusion geometries

**Fig. 2:**
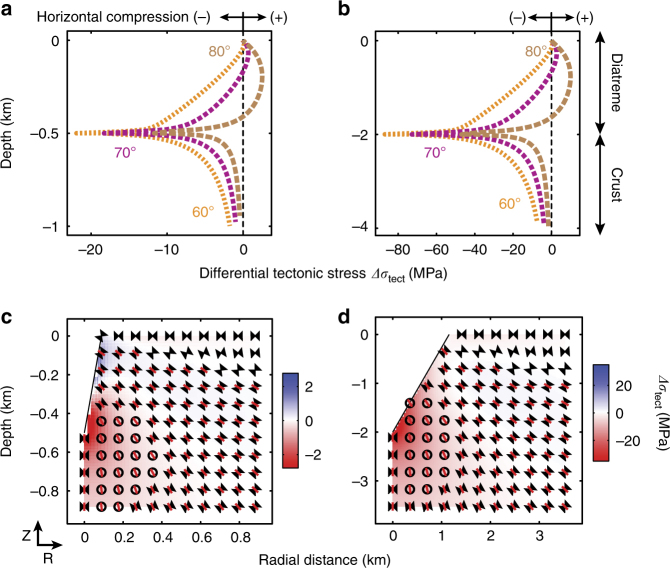
State of stress within the crust surrounding the newly formed diatreme. **a**, **b** Differential tectonic stress along the diatreme flank and in the underlying crust for different wall dips (*α* = 80, 70, and 60°, in brown, purple, and orange-colored thick-dashed lines, respectively) and for different diatreme depths *D*_d_ = 500 m (**a**), *D*_d_ = 2000 m (**b**). Separated by a dash line, positive and negative values correspond to horizontal extensional and compressional stresses, respectively. **c**, **d** Differential tectonic stress and stress orientation within the crust for different diatreme geometries, *D*_d_ = 500 m, *α* = 80° (**c**); *D*_d_ = 2000 m, *α* = 60° (**d**). Blue and red shadings represent extensional and compressional *Δσ*_tect_, respectively. Based on Melosh and Williams^34^ and McGovern and Solomon^35^, the hourglass shapes are oriented along the direction of greatest compressive stress (*σ*_1_); the red bars along the direction of least compressive stress (*σ*_3_). Circles represent out-of-the plane hourglass shapes (*σ*_1_ perpendicular to the r–z plane)

We focus our analysis on the differential tectonic stress state

1 $$\Delta \sigma _{\mathrm{tect}} = \sigma _{\mathrm{r}} - \sigma _{\mathrm{z}},$$

with *σ*_r_ being the radial (horizontal) stress and *σ*_z_ the vertical stress, created within the crust and the diatreme infill, which affects the driving forces for vertical magma movements^25,26^. Negative values correspond to environments under horizontal compression (*σ*_z_ > *σ*_r_) (e.g., in red Fig. 2c), which discourages vertical magma propagation in dikes and promotes lateral propagation in sills^27^. Positive values correspond to areas under horizontal extension (*σ*_r_ > *σ*_z_), which favor vertical magma propagation (e.g., in blue Fig. 2c).

**Fig. 3:**
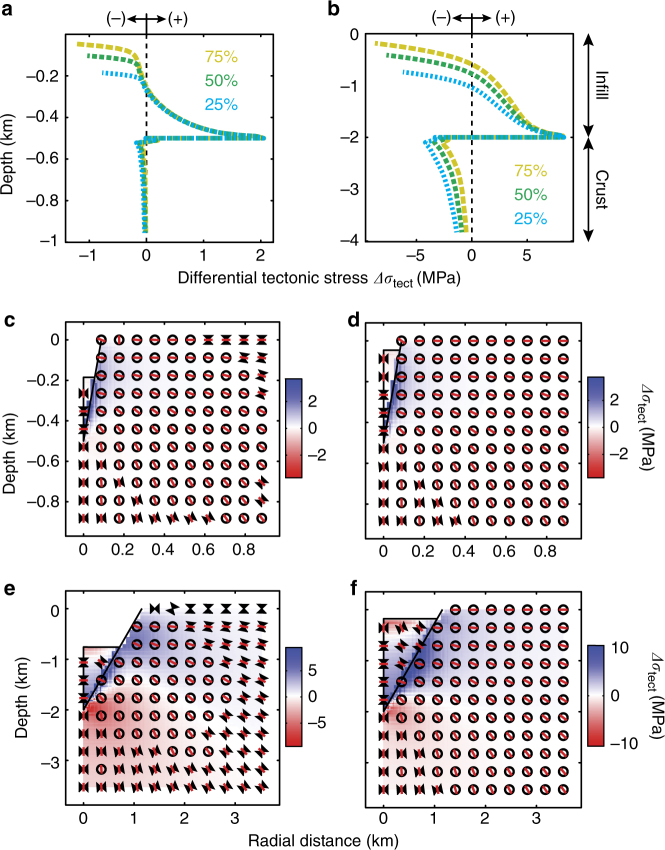
State of stress within the diatreme’s infill and the surrounding crust. **a**, **b** Differential tectonic stress along the symmetrical axis of the model (left boundaries) for different diatreme’s depth *D*_d_ = 500 m (**a**), *D*_d_ = 2000 m (**b**), dipping angles *α* = 80° (**a**), *α* = 60° (**b**), and infill volume (colors blue, green, and yellow for 25, 50, 75% of infill, respectively). **c**–**f** Differential tectonic stress and stress orientation within the infill and the crust for different diatreme geometries and infill volumes, **c** *D*_d_ = 500 m, *α* = 80°, 25% infill; **d** *D*_d_ = 500 m, *α* = 80°, 75% infill; **e** *D*_d_ = 2000 m, *α* = 60°, 25% infill; **f** *D*_d_ = 2000 m, *α* = 60°, 75% infill. Legend as in Fig. 2

Finally, the orientation of the minimum compressive stress (*σ*_3_) is inferred to indicate the type of magmatic intrusion occurring within the domain, assuming that magmatic intrusions open perpendicular to *σ*_3_ (red bars in Figs. 2, 3)^25^. In regions subjected to horizontal extension, *σ*_3_ may be oriented in any direction along the horizontal plane and symmetry axis. Magma transport occurs through planar intrusions^25^, such as dikes (dips >60°) and sills (dips <10°), and our models and figures show a cross-section normal to the long-axis of a hypothetical intrusion. Other secondary factors known to affect intrusion geometries, such as preexisting structures^10^ and layers with contrasting mechanical properties^9,28–31^, were not considered in our modeling approach, but if included would further promote the development of the complex intrusive networks discussed^9,32^.

### Stress states during diatreme excavation and infilling

Excavated diatreme structures (no infilling) with steep diatreme walls (80° dip), subjected to instantaneous unloading, exhibit horizontal extension in the upper ~90% of the diatreme walls (positive values and blue shading in Fig. 2a, c, and Supplementary Figs. 1, 2), while the lower part, as well as the underlying host rock, are subjected to horizontal compression (negative values and red shading in Fig. 2a, c). Diatremes with gentler slopes show a decrease in the area subjected to horizontal extension (e.g., from ~40% of the wall for 70° dips to 0% for 60° dips) and an increase in the area of horizontal compression in the lower part of the diatreme and underlying crust (Fig. 2a, b, and Supplementary Figs. 1, 2). In all, we observe differential tectonic stresses down to −80 MPa in areas of horizontal compression, and up to 10 MPa in areas of horizontal extension in the country rock surrounding excavated diatremes with no infilling (Supplementary Fig. 1). The orientation of *σ*_3_ is vertical below the excavated diatreme, favoring sill formation, but rotates in the illustrated vertical r–z plane of the model in an anticlockwise manner to become sub-horizontal in the surrounding crust, which favors dike formation (red lines Fig. 2c, d and Supplementary Fig. 2). Finally, *σ*_3_ rotates further, immediately below the ground surface, into an orientation out of the illustrated plane (hourglasses without red lines, Fig. 2c, d and Supplementary Fig. 2).

Immediately following a major diatreme-excavation explosion, the diatreme structure is partly re-filled with vertically ejected volcaniclastic material. These infills exhibit horizontal extension of up to 10 MPa at their base. Maximum horizontal compression observed in the upper half of the infill is dependent on the infill volume percent and ranges from −1 to 10 MPa in our model runs (Fig. 3a, b and Supplementary Fig. 3). These stress magnitudes are also sensitive to the Young’s Modulus of infill, although stress orientations remain constant (see Supplementary Figs. 5–8). In the country rock below the diatreme, horizontal compression is present and its magnitude is also dependent on the infill volume percent and Young’s Modulus of the infill material. Values below the diatreme range up to −4 MPa (Fig. 3a, b and Supplementary Fig. 3). Horizontal extension occurs near the contact between the diatreme wall and the infill, with differential tectonic stress values up to 10 MPa (Fig. 3b). The orientation of *σ*_3_ within the infill is horizontal in its lower half, favoring dike intrusion, and vertical in the upper half, favoring sill intrusion. Within the surrounding host rock, the orientation of *σ*_3_ is vertical below the diatreme and rotates to sub-horizontal in the vicinity of diatreme walls up to the surface (Fig. 3c–f, Supplementary Figs. 9–12).

### Impact on intrusive processes and magma fragmentation

Maar-diatreme formation results from country rock excavation and remobilization during explosive volcanism. A long-standing model of maar-diatreme formation involves progressive deepening of the base of the diatreme as the site of magma fragmentation descends due to water table drawdown^33^. By contrast, recent field, experimental, and numerical studies support fragmentation zones at almost any location and depth during diatreme formation^13,36–39^. The progressive up-structure widening of the diatreme in the latter models results from collapse of unsupported wallrock^38^, explosive fragmentation at shallower depths^37^, and also from lateral magma diversions, possibly in sills in the upper diatreme^40^. Such models thus require that vertically ascending magma preferentially stalls in the shallow subsurface, which our model shows is favored by the stress response to infilling.

Comparing natural volcanic features with products of theoretical models is fundamental for understanding the physical processes that influence the nature of volcanic activity and growth of different volcano types. In the shallow subsurface, dike overpressures typically range 1–10 MPa^41,42^. This overpressure is focused within the dike’s head, and is often released upon explosive fragmentation, with subsequent magma arriving from the dike tail being only weakly overpressured^43^. Local increases in horizontal compressive stresses of a few MPa tend to inhibit magma ascent in subvertically oriented dikes; in these instances, intrusion propagation may terminate entirely, or dikes will reorient according to the new stress state^27^, or intrude along crustal heterogeneities^44^. Field observations in monogenetic fields support stress changes that alter directions of intrusion propagation. For example, dikes are shown to transform to sills near the base of^3^, or within^40^, diatremes, and saucer-shaped sills (see Muirhead et al.^3^ for a detailed description) outcrop at the peripheries of nested diatremes and volcanic–conduit complexes^3^ (Supplementary Figs. 13, 14).

**Fig. 4:**
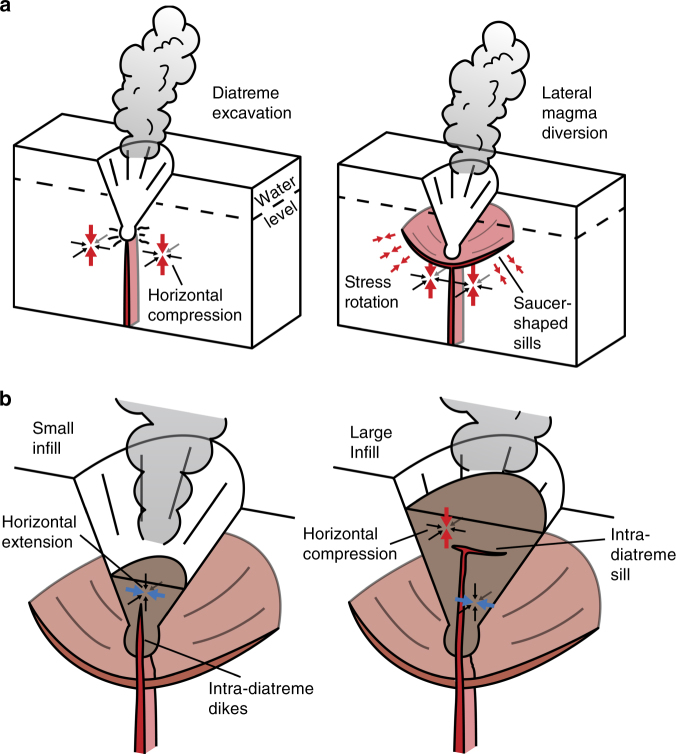
Sketch of a maar-diatreme eruption and formation of the magmatic plumbing system. **a** The proto-diatreme (aka excavation stage) and **b** developing diatreme (aka infilling stage). The colored arrows represent the orientation of the minimum compressional stress (*σ*_3_), the blue and red colors represent the differential tectonic stress, extensional and compressional, respectively

By examining a simplified two-step process of maar-diatreme formation (excavation and infilling), our models reveal stress variations that explain these observed lateral magma diversions during explosive maar-diatreme volcanism. The initial process of diatreme excavation leads locally to unloading of the crust, and horizontal compression is generated in the crust underlying the newly formed diatreme, which results in a local stress rotation and a subvertical minimum compressional stress (*σ*_3_). Such a state of stress inhibits the vertical propagation of magma and favors lateral diversions through sills near the base of the diatreme (Fig. 4a). Similar magma diversions are shown to result from vertical unloading of the crust during rifting^11^. The tendency for a compressional stress state to induce dike arrest and lateral magma diversions will be greater for dikes with relatively low overpressures (no more than a few MPa)^45^, as is expected for mafic monogenetic eruptions, which exhibit relative low magma volumes and fluxes^10^. Inelastic processes, such as fracturing will occur during the initial explosive process. The damage zone would decrease the magnitude of the stress within the medium, but would not change the main characteristics of the horizontal and extensional stress^46^.

During the second step, as the diatreme is filled with volcaniclastic material, a reduction in the magnitude of horizontal compression below the diatreme allows magma to ascend in dikes into the diatreme. For a range of plausible infill properties (sensitivity tests in Supplementary Figs. 4–8), horizontal extension generated in the lower half of the diatreme infill will further promote the upward ascent of dikes in these areas, as shown in nature by dikes observed in the lower diatreme (Fig. 4b) (e.g., ref. ^39^ and references therein). As predicted by Valentine and White^36^, our results show that the orientation and magnitude of *σ*_3_ within the infill can also promote irregular magma propagation paths (sub-vertical to inclined), and that magma can be drawn to the peripheries of the diatreme, which experience the greatest horizontal extensional stresses. Finally, once a significant volume of diatreme infill has been emplaced, horizontal compression is generated in the subsurface of the infill, stalling ascending magma and promoting magma-host mingling or lateral diversion of magma into sills (Fig. 4b). Once a laterally propagating sill reaches areas of horizontal extension near the diatreme flanks, it can then transform back into a dike or inclined sheet (dipping 10–60°^3^), ultimately producing a saucer-shaped sill geometry^3^. Additionally, as the laterally propagating sill in the upper diatreme reaches the diatreme walls magma will enter an area of horizontal extension that favors groundwater entry and thus phreatomagmatic explosions, resulting in upward widening of the overall structure^36^ and explosive surface eruptions.

Finally, as the diatreme develops and both the depth and volume of the clastic infill increase, the magnitude of shallow horizontal compression in the upper diatreme also increases. This is expected to have two important effects. First, increased horizontal compression in the upper part of the diatreme decreases the likelihood that dikes will reach the surface, thus requiring greater magma flux rates and overpressures to drive eruptions. Therefore, larger explosions would be predicted in evolved diatreme structures, as described by Valentine and White^36^. Secondly, lateral diversions of magma are expected to become more frequent within larger diatremes, and thus sill-driven lateral quarrying will be favored in well-developed examples, which may assist in the formation of nested diatremes^47^.

In conclusion, diatreme formation is a complex process that occurs during kimberlite and basaltic phreatomagmatic eruptions^48,49^. Furthermore, excavation of vents, with or without infilling, occurs during explosive eruptions of other styles (e.g., vulcanian and plinian eruptions). The stress changes examined here are expected also to take place in a variety of volcanic settings and eruptive scenarios, including on other planetary bodies, though typically imposed on different pre-eruption topography. For instance, syn-eruptive sill transitions and shallow magma diversion have been recorded seismically and geodetically at composite volcanoes (e.g., Stromboli, Italy^50^), and sill/laccolith inflation was detected after the onset of explosive volcanism during the 2011 eruption of Cordón Caulle, Chile^12^. Here, we show that stress states resulting from explosive excavation of country rock and diatreme infilling provide a novel mechanism to explain sill transitions and saucer-shaped sill formation in the crust immediately enclosing diatremes. Modeled stress fields in this study, and resulting intrusion geometries, provide new insights into processes controlling diatreme development, and support recent models of diatreme growth proposed by Valentine and White^36^ and Valentine et al.^39^. Overall, modeled stress states reveal a critical feedback between explosive maar-diatreme vent excavation, infilling, and development of geometrically complex magma networks. Phases of explosive excavation encourage magma to stall in sills below the excavated structure, allowing for lateral changes in the position of fragmentation zones early in the diatreme’s history rather than progressive deepening, whereas subsequent infilling should promote magma ascent to shallower levels within the diatreme. Horizontal compression in the upper diatreme drives lateral magma diversions, encouraging explosive diatreme widening and growth at shallow depths. Compressional stresses resulting from increasing infill volumes during continued diatreme growth are expected to promote sill-driven lateral quarrying, nest-diatreme formation, and larger explosive activity in well-developed examples. Our results suggest that while the diversion of magma during hazardous explosive eruptions may interrupt the eruptive sequence it does not necessarily indicate the end of an eruption, and can presage a shift in the vent location^6,8^. Such vent-site shifts should be considered in volcanic hazard mapping and eruptive-response scenarios^19^; detection of shifts and their precursors can be improved through more intensive geophysical and geodetic monitoring^12,51^.

## Methods

### Axisymmetric finite element modeling

We use finite element models through COMSOL Multiphysics® to investigate for the first time the effects of diatreme excavation for single-explosion craters followed by the infilling of the newly formed diatreme on the local state of stress (Fig. 1). These stress changes are expected to affect the geometry of surrounding intrusions, which form normal to the least compressive stress^52^. Our approach allows us to calculate in 3D the locations favoring or not the vertical propagation of magmas, as well as the stress orientation controlling the geometry and direction of propagation of magmas. One limitation of finite element studies testing a 3D stress environment is that it limits the ability to model active intrusion propagation^23^, which currently can be performed only in a 2D stress state. However, combined numerical and analog studies show that intrusion propagation paths closely mimic those predicted by the modeled 3D stress state^53^.

### Host rock state of stress

The 2D axisymmetric elastic domain has a Young’s Modulus (*E*_c_) of 15 GPa (sandstone equivalent), a density (*ρ*_c_) of 2300 kg m^−3^, and Poisson’s ratio (*µ*) of 0.25. The area of the elastic domain is 100 × 100 km to avoid side effects, and is subjected to gravitational loads^54^ expressed by an initial lithostatic stress

2 $$\sigma _{\mathrm{r}} = \sigma _{\mathrm{\theta }} = \sigma _{\mathrm{z}} = - \rho _{\mathrm{c}}.\,Z.\,g,$$

where $Z$ is negative downward and negative stress values indicate compression, and $g$ is the Earth’s gravitational acceleration (−9.81 m s^−2^), and a body load

3 $$\rho _{\mathrm{c}}.\,g$$

### Excavation stage

The diatremes were initially modeled as inverted cones corresponding to an excavated volume of rock, where the depth to base of the diatreme and angle of the diatreme walls varied from 500–2000 m and 60–80°, respectively (Supplementary Table 1). An unloading force representing the missing vertical rock load is applied at the surface of the diatreme (red line in Fig. 1b)^11^. This represents an end-member situation in which a deep open crater forms prior to any infilling, as has been inferred for some kimberlites^55^.

### Infilling stage

The second step of each model tested the effects of infilling of the diatreme after excavation (Fig. 1c). Their initial conditions (deformed geometry and state of stress) corresponded to the transferred solution of the initial model using COMSOL capabilities. Here, we consider the static case when the infill material is fully deposited, and hence not in a fluidized state^56^. The diatreme was filled with a lower-density volcaniclastic material (*ρ*_i_ = 2000 kg m^−3^), which exhibited a weak elastic response (*E*_i_ = 2.5 GPa, and *µ* = 0.25) compared to the surrounding, consolidated country rock. Sensitivity tests conducted for these parameters are presented in the Supplementary Material. These sensitivity tests reveal that similar stress orientations are generated within and around diatremes for a reasonable range of elastic moduli (i.e., 0.5–5 GPa). To test the varying effects related to the weight of the infill, a series of model runs were performed with the initial excavated cavity filled by 25, 50, and 75% of its original volume. We make the simplifying assumption that the material is deposited en masse^57^. Body loads were added to the infilling as well as the host rock to simulate the gravitational load. The contact between the infill and the host rock is not fixed, which allows the diatreme fill to deform independently of the surrounding domain.

### End-member scenarios

Our models address end-member scenarios, and real diatreme-forming eruptions are expected to have more complex excavation and infilling histories in which the diatreme is only partially emptied or infilled^13,38^. Nevertheless, the “forcing directions” of the effects of excavation vs. infilling of the diatreme structure revealed by our modeling show the potential for country-rock responses to modulate magma behavior during evolution of diatremes.

### Results from numerical modeling experiments

A parametric analysis to study the role of the geometry and size of the diatreme has been implemented during this study (Supplementary Table 1 and Supplementary Figs. 1–3 and 9–12).

### Model sensitivity to volcaniclastic infill properties

The creation of a diatreme implies deposition within the diatreme structure of material resulting from the fragmentation of the country rocks and the magma. The sequence of events during the initial excavation and the filling of the diatreme is complex. While the material is believed to be deposited en masse^57^, it can remain fluidized^58^ until it is fully deposited. The nature of the deposit is a mixture of both consolidated and granular material, which are expected to behave either elastically or viscously, respectively (e.g., tuff, lapilli-tuff, and non-volcanic sediments mixed with basaltic magma^59–61^). The physical characteristics of the infill are a function of particle size range and packing along with other factors (e.g., moisture/water content), and encompass a wide range of values. The rheological behavior of the diatreme’s infill will therefore vary depending on the degree of rock induration at the time of intrusion, which will be strongly dependent on (1) the time interval between excavation and new magma intrusion events, (2) sediment properties affecting porosity (i.e., sorting, size, shape)^62^, and (3) the depth of the diatreme fill. As such, diatreme infills exhibit a variety of structures, such as brittle faults and intrusions (dike, sills)^40^, non-brittle folds^ 63^, and peperite^60^, which reflect elastic to elastoplastic to fluid-like viscous behaviors.

To account for varying elasticity, we run a series of models that test a range of elastic moduli values, with some values acting to suppress the elastic response of the diatreme fill. We tested the sensitivity our models to a low and high density material (1500 kg m^−3^ instead of 2000 kg m^−3^ as in Gernon et al.^58^, and 2500 kg m^−3^, Supplementary Fig. 4) and Poisson’s ratio (0.2 and 0.3 instead of 0.25, Supplementary Fig. 4), and a variety Young’s modulus values (0.5, 1, 2.5, and 5 GPa instead of 10 GPa, Supplementary Figs. 5–8).

Results show that density and Poisson’s ratio have a negligible effect on the resulting stress magnitudes (Supplementary Fig. 4). We observe that only the infill density influences the magnitude of the differential tectonic stress. Lower-density values favor larger horizontal compressional stresses in the underlying crust and lower differential tectonic stress within the infill, and vice versa for larger densities (Supplementary Fig. 4). Although Young’s modulus does not influence stress orientations, it does affect the magnitudes of stresses sustained within the domain. In all, a decrease in Young’s modulus lowers the amount of stress generated within the diatreme fill, as decreasing this value gradually suppresses the elastic response of the material (Supplementary Figs. 5–8). However, stress magnitudes even for low values of Young’s modulus are still high enough to promote the magma diversions discussed in this study. Furthermore, lowering Young’s modulus actually increases the magnitudes of compressional stresses immediately below the base of the diatreme, and thus lateral magma diversions in sills are still expected during crater-forming eruptions even if elasticity is completely suppressed within the diatreme fill.

Although Young’s modulus is a relatively unknown parameter for the diatreme fill, these sensitivity analyses validate our approach and results, because the principle stresses remain the same between models, and thus intrusion geometries and the directions of magma diversions are consistent for the full range of reasonable elastic moduli values. Secondly, the magnitudes of the stress changes below and within the diatremes are high enough (always greater than a few MPa) to promote magma diversions.

### Field observations of lateral magma diversion in maar-diatreme fields

Recent field and seismic reflection studies have documented lateral magma diversions, particularly in sills, related to monogenetic volcanoes^1,3,5,22,40,64–66^. The mechanical controls on sill formation in these fields are poorly constrained, and little is known regarding the important feedbacks between intrusion and eruptive processes during eruptive episodes. Re et al.^22^ and Muirhead et al.^3^ suggested that saucer-shaped sills observed in the Hopi Buttes volcanic field possibly formed in response to subaerial and/or subsurface volcanic additions/removals of mass. Re et al. [22] hypothesized that the presence of a volcanic load (e.g., a scoria cone) could produce local stress rotations immediately below volcanoes at Hopi Buttes volcanic field, which could lead to sill formation. However, it is unknown whether scoria cones were present during emplacement the intrusions documented in their study.

Model results presented in this study provide new insights into how these observed sills, and lateral magma diversions generally, may be dynamically linked with the excavation and filling of diatremes and other volcanic conduit structures during explosive eruptive episodes. Indeed, in the Hopi Buttes volcanic field, clear spatial associations are observed between sills, diatremes, and pyroclastic vent structures (termed massifs by ref. ^67^). The presence of bedded, vesicular pyroclasts (scoria) in these vent structures suggests that at one time they formed open cavities exposed at the surface. Below these vents, sub-vertical dikes are observed transitioning into sub-horizontal sills (stage a of Fig. 4). The geometrical arrangement of segments and orientation of segment long-axes support magma propagation radially away from these central conduits in saucer-shaped sills^3^. At Crown Butte, saucer-shaped sills surround two (nested?) maar-diatremes. These sills occur at upper diatreme levels, as predicted for a developing diatreme with large infill (stage b of Fig. 4), and primary magma flow indicators (i.e., orientation of segment long-axes) suggest magma has flow laterally away from within or below these diatremes (Supplementary Fig. 14)^3^. In all, these observations support magma diversions in sills during excavation and filling stages of diatreme development, and at different depths in the diatreme complex as predicted by the numerical modeling results.

### Data availability

All the relevant data that have been used in the present study are available from the authors.

## Electronic supplementary material

Supplementary Information

Peer Review Report
