# Supplementary material for: Shallow magma diversions during explosive diatreme-forming eruptions
Source: Nat Commun. 2018 Apr 13;9:1459. doi: 10.1038/s41467-018-03865-x (PMC5899137; doi:10.1038/s41467-018-03865-x)
Supplement: Supplementary file 1 — Supplementary Information [file 41467_2018_3865_MOESM1_ESM.pdf]

Supplementary Material for

Shallow magma diversions during explosive diatreme-forming eruptions

Nicolas Le Corvec, James D. Muirhead, and James D. L. White

R_d (m)	D_d (m)	α (°)	Volume (m^3)
88	-500	80	4.05E+06
182	-500	70	1.73E+07
289	-500	60	4.37E+07
176	-1000	80	3.24E+07
364	-1000	70	1.39E+08
577	-1000	60	3.49E+08
264	-1500	80	1.09E+08
546	-1500	70	4.68E+08
866	-1500	60	1.18E+09
353	-2000	80	2.61E+08
728	-2000	70	1.11E+09
1155	-2000	60	2.79E+09

Supplementary Table 1: Geometrical characteristics the diatremes' sizes. R_d, D_d and α represent the diatreme radius, depth, and dip, respectively.

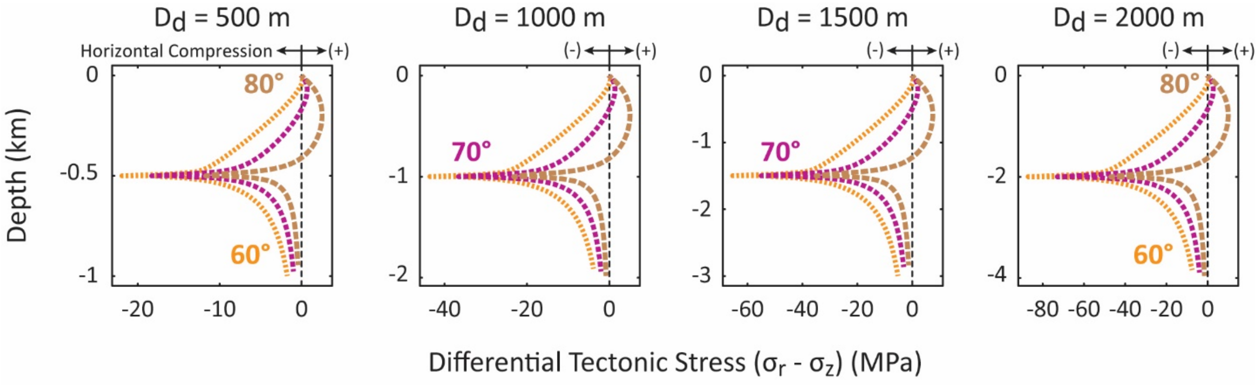

Supplementary Figure 1: Differential tectonic stress along the diatreme's wall and in the underlying crust. D_d represents the diatreme depth. The colored values represent the dip angle of the diatreme's wall. Legend as in Fig 2.

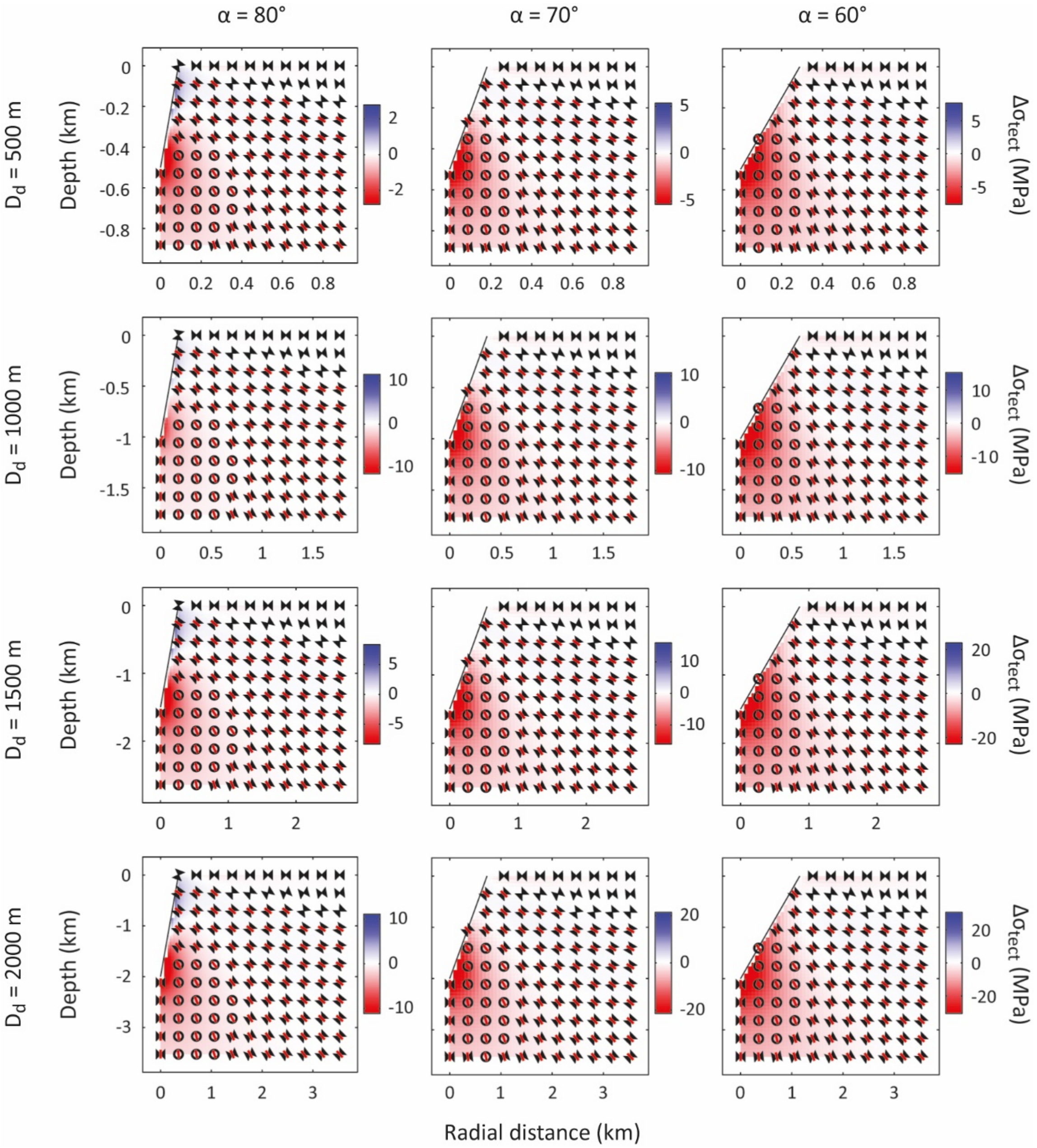

Supplementary Figure 2: State of stress within the surrounding crust during the excavation stage. Different diatreme's depths (D_d) and different dipping angles (α) were tested. Legend as in Fig. 2.

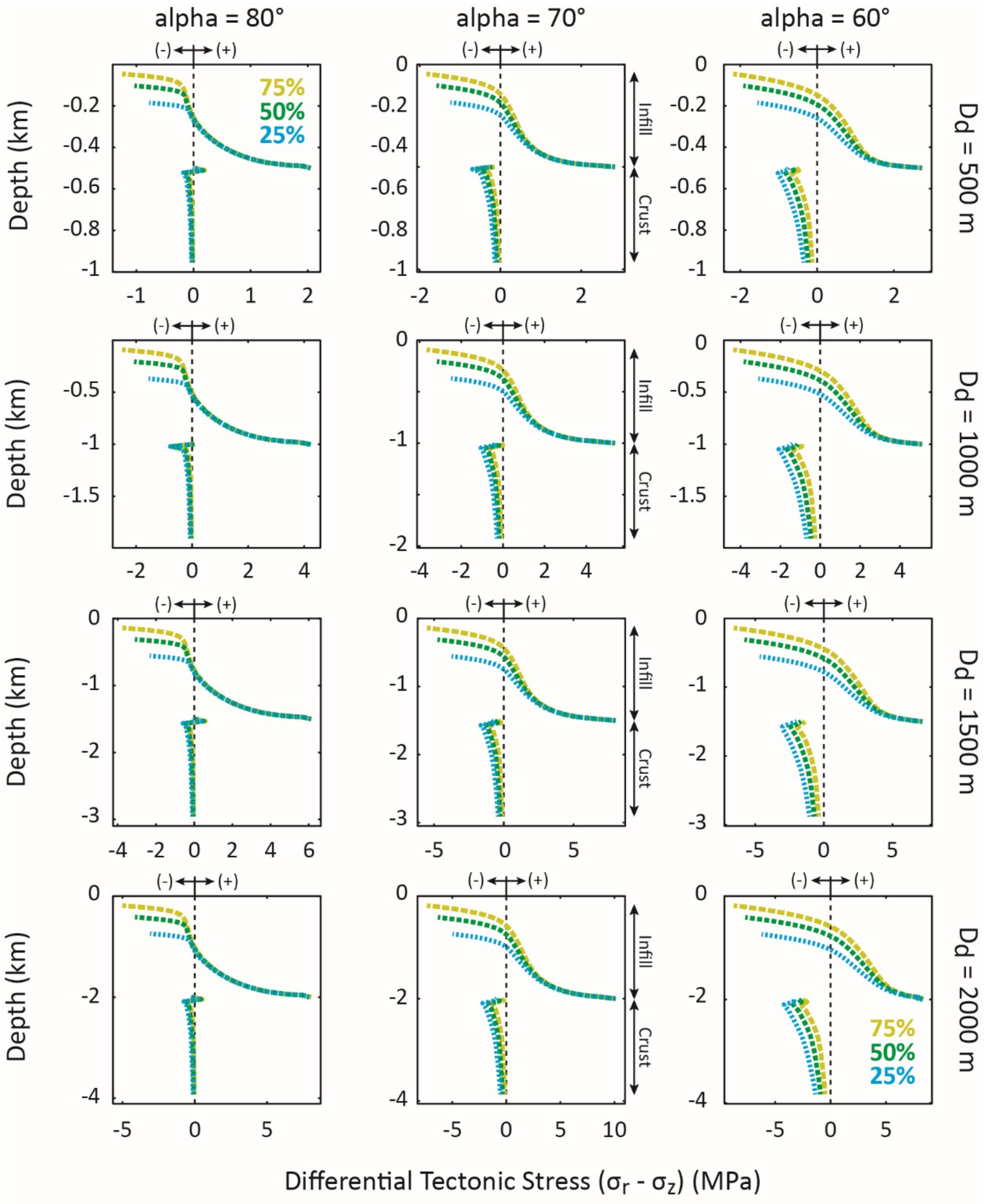

Supplementary Figure 3: State of stress within the diatreme's infill and within the surrounding crust. a and b) Differential tectonic stress along the symmetrical axis of the model (left boundaries) for different diatreme's depth (D_d), dipping angles (α), and infill volume (colors blue, green and yellow for 25, 50, 75% of infill, respectively).

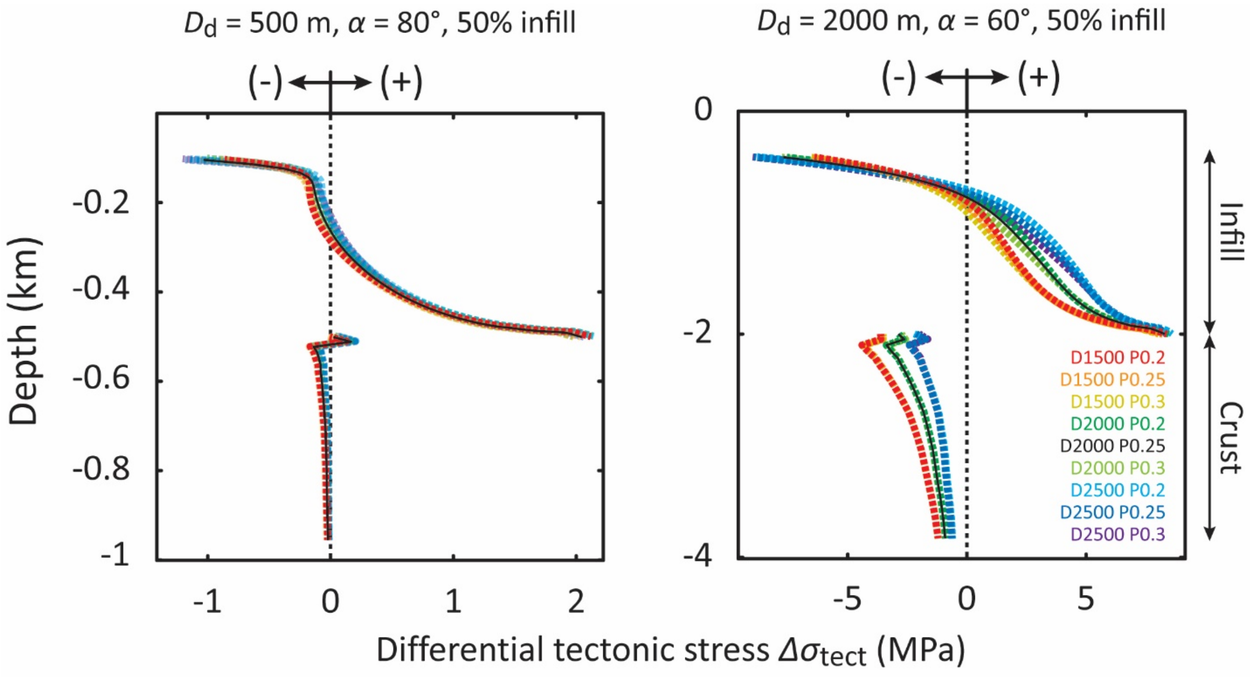

Supplementary Figure 4: Infill's elastic parameters sensitivity test. Different Poisson ratios and densities were tested for the infill of a shallow and a deep diatreme (500 and 2000 m deep, respectively) filled with 50% of their initial volume and with a dip angle (α) of 60°. Young's Modulus infill = 2.5 GPa. Density values (D) range from 1500 to 2500 kg m^{-3} and Poisson's ratio (P) values range from 0.2 to 0.3. Colors represent different density and Poisson ratio values. Positive differential tectonic stress ($\Delta\sigma_{tect}$) represents areas under horizontal extensional stress; negative values represent areas under horizontal compressional stress.

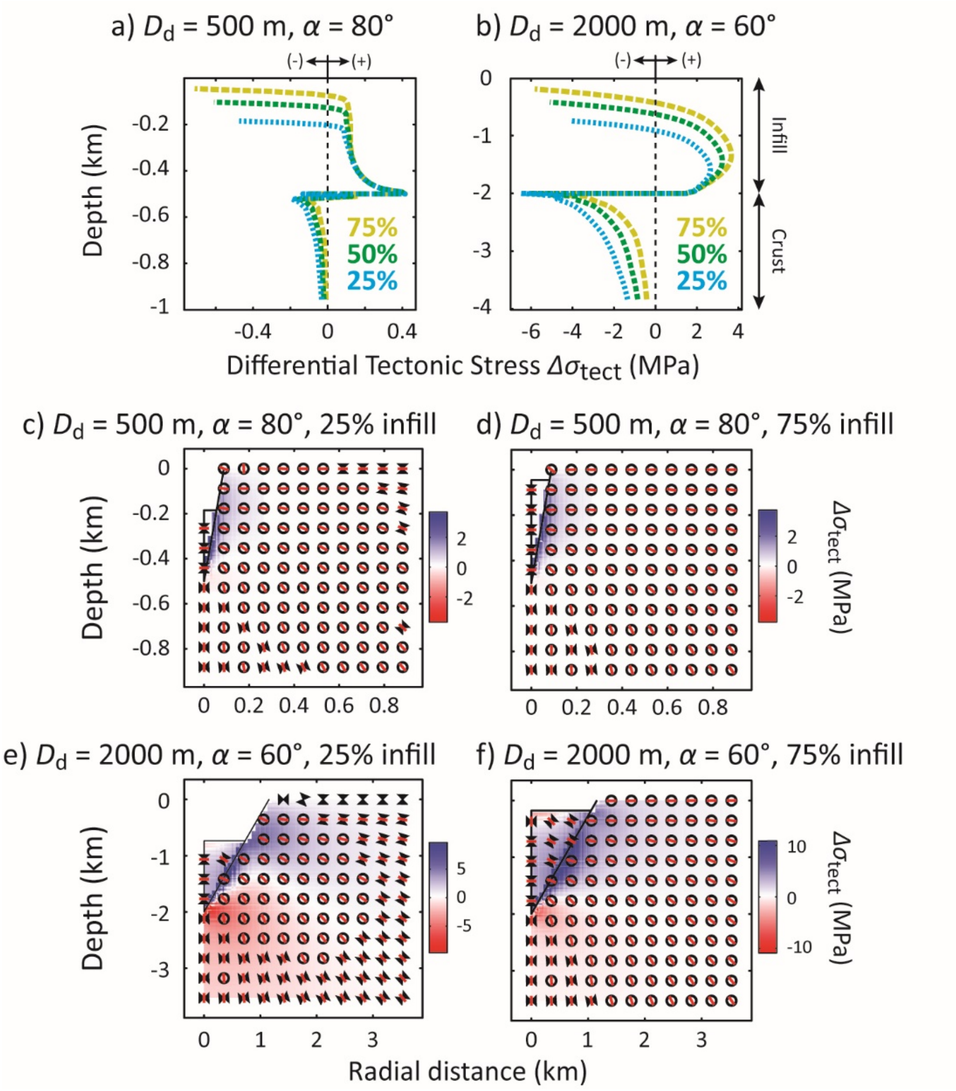

Supplementary Figure 5: State of stress within the diatreme's infill and within the surrounding crust. Young's Modulus infill = 0.5 GPa. a and b) Differential tectonic stress along the symmetrical axis of the model (left boundaries) for different diatreme's depth (D_d), dipping angles (α), and infill volume (colors blue, green and yellow for 25, 50, 75% of infill, respectively). c to f) Differential tectonic stress and stress orientation within the infill and the crust. Legend as in Fig. 2.

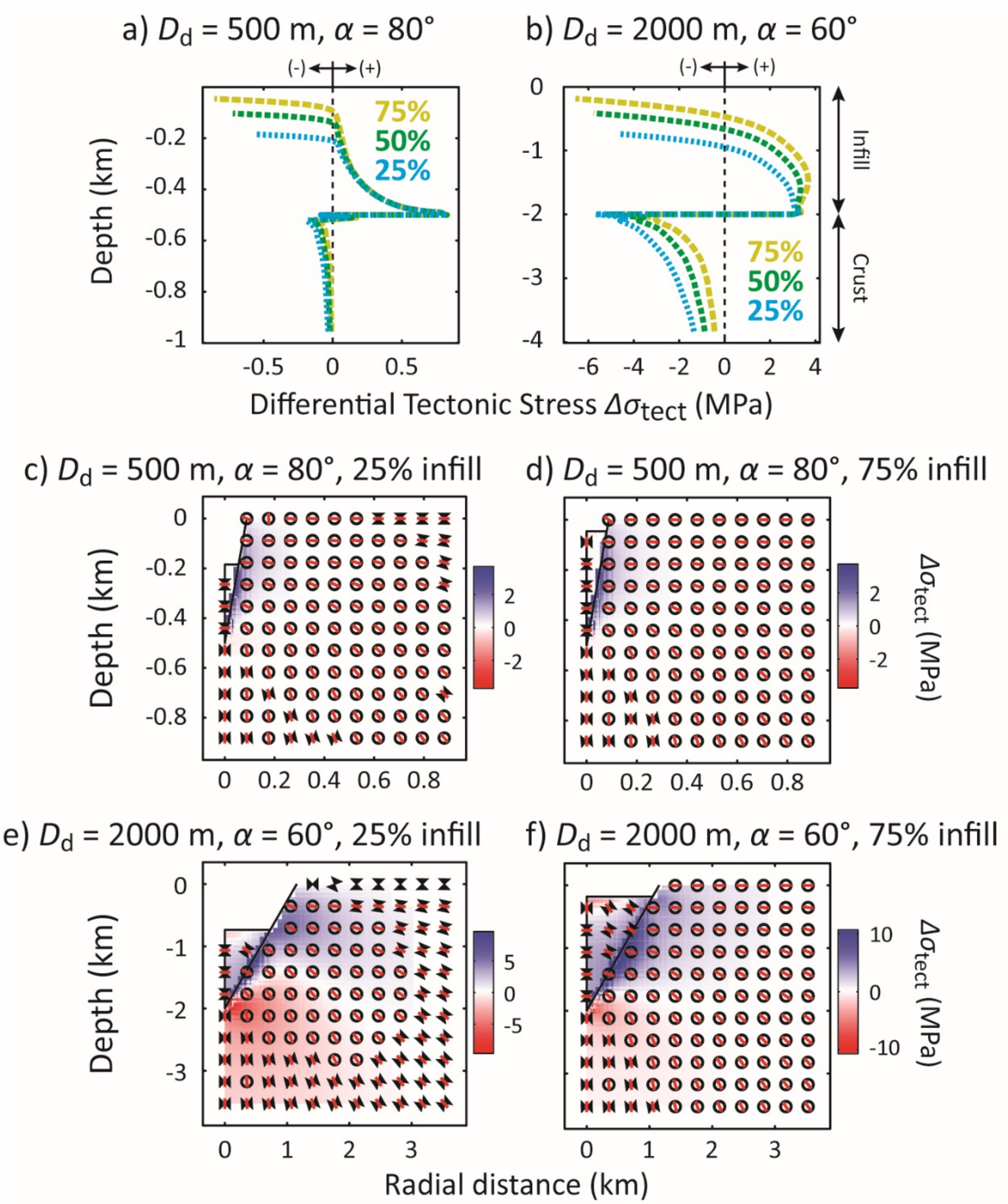

Supplementary Figure 6: State of stress within the diatreme's infill and within the surrounding crust. Young's Modulus infill = 1 GPa. a and b) Differential tectonic stress along the symmetrical axis of the model (left boundaries) for different diatreme's depth (D_d), dipping angles (α), and infill volume (colors blue, green and yellow for 25, 50, 75% of infill, respectively). c to f) Differential tectonic stress and stress orientation within the infill and the crust. Legend as in Fig. 2.

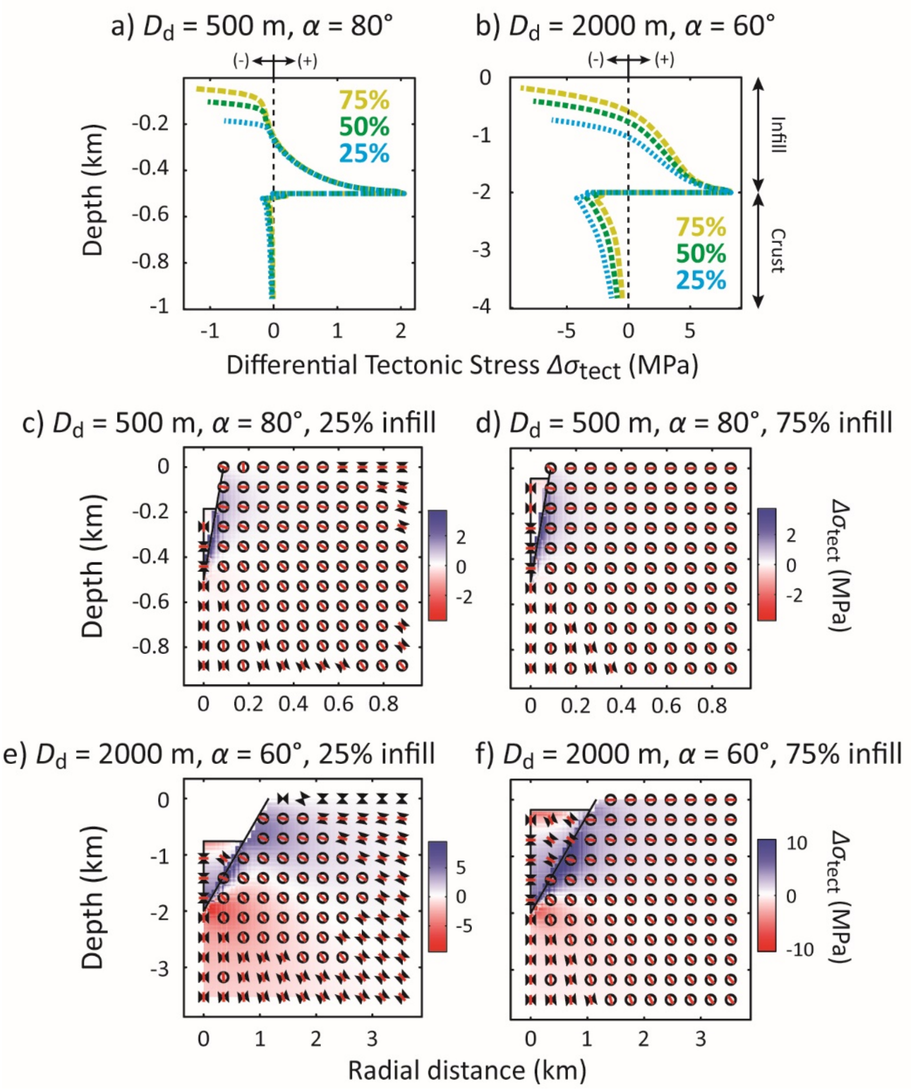

Supplementary Figure 7: State of stress within the diatreme's infill and within the surrounding crust. Young's Modulus infill = 2.5 GPa. a and b) Differential tectonic stress along the symmetrical axis of the model (left boundaries) for different diatreme's depth (D_d), dipping angles (α), and infill volume (colors blue, green and yellow for 25, 50, 75% of infill, respectively). c to f) Differential tectonic stress and stress orientation within the infill and the crust. Legend as in Fig. 2.

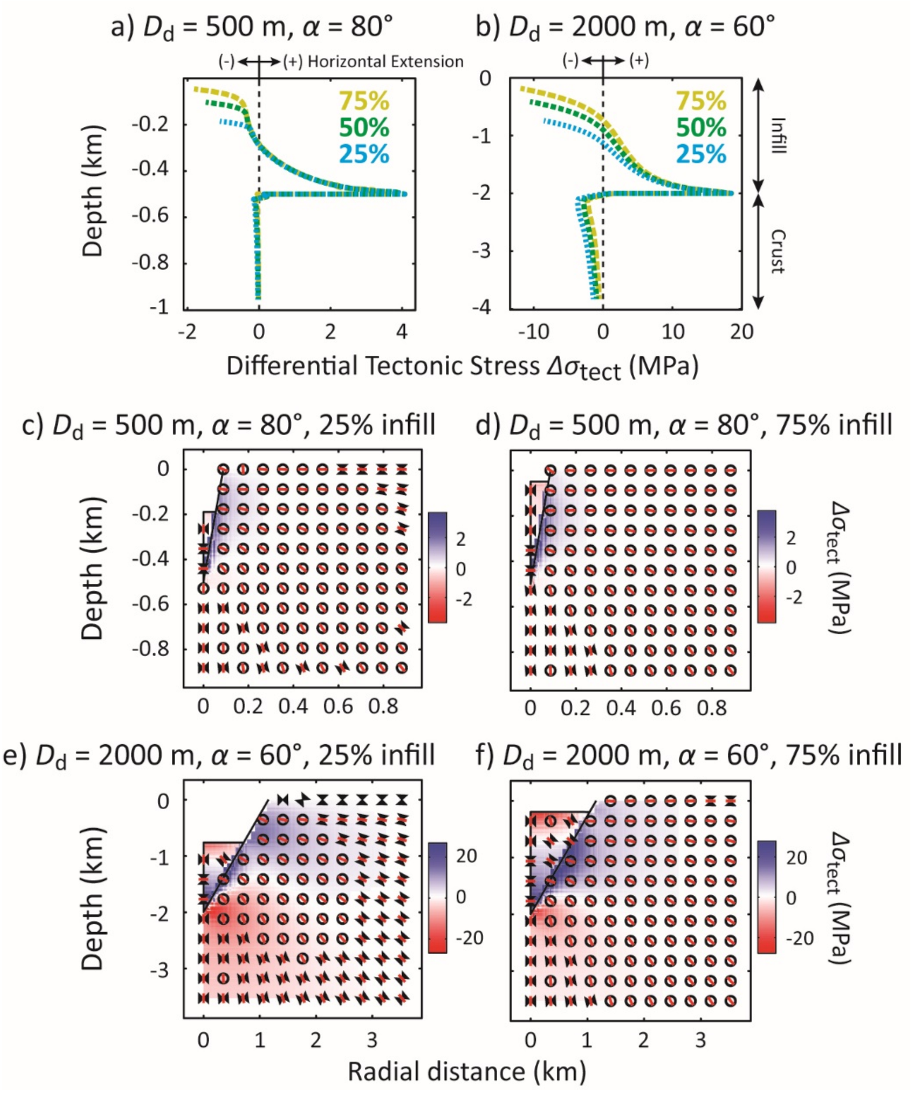

Supplementary Figure 8: State of stress within the diatreme's infill and within the surrounding crust. Young's Modulus infill = 5 GPa. a and b) Differential tectonic stress along the symmetrical axis of the model (left boundaries) for different diatreme's depth (D_d), dipping angles (α), and infill volume (colors blue, green and yellow for 25, 50, 75% of infill, respectively). c to f) Differential tectonic stress and stress orientation within the infill and the crust. Legend as in Fig. 2.

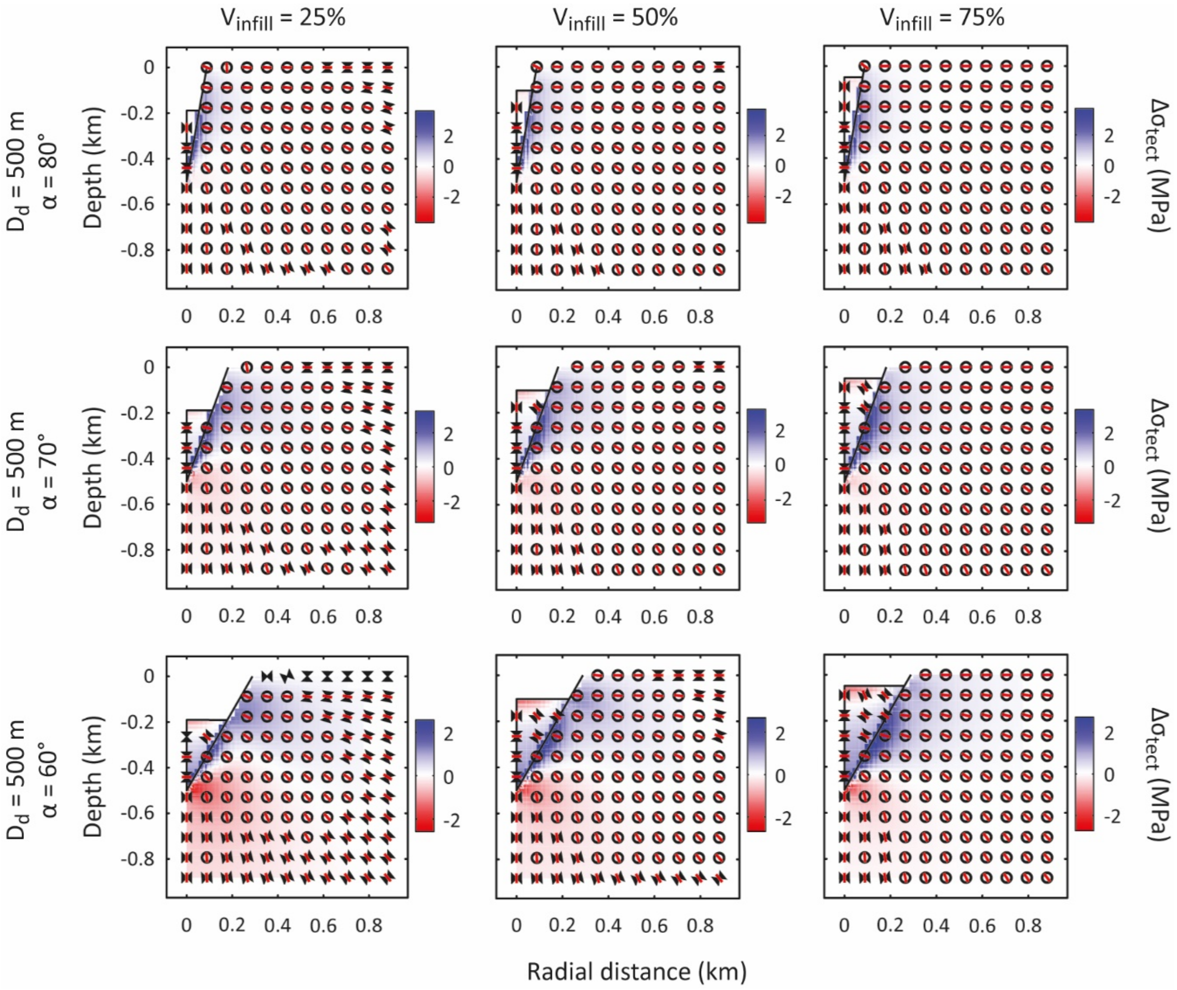

Supplementary Figure 9: State of stress within the diatreme's infill and the surrounding crust. Diatremes with a depth (D_d) of 500 m and with different dipping angles (α) and infill percentages (V_{infill}). Legend as in Fig. 2.

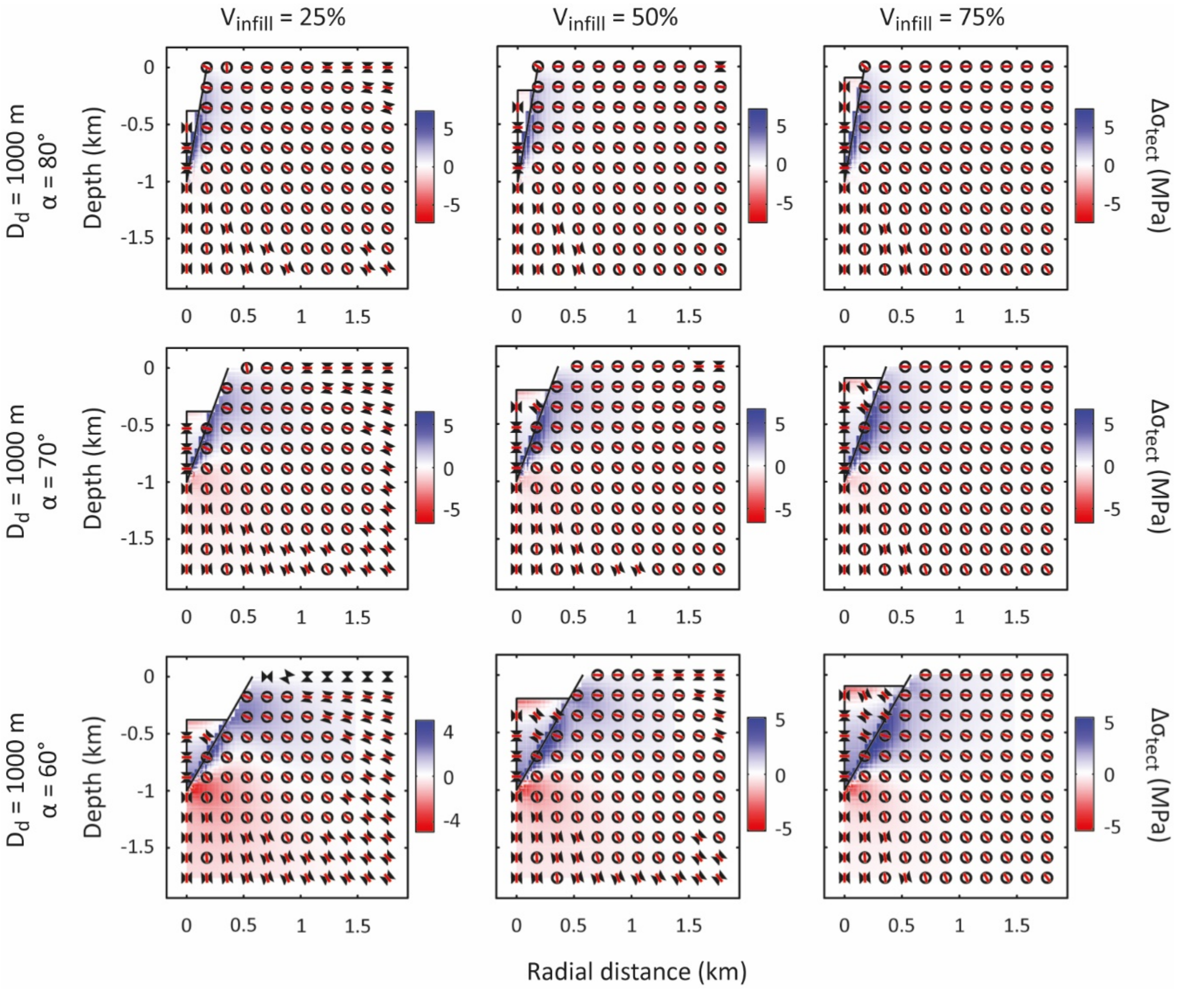

Supplementary Figure 10: State of stress within the diatreme's infill and the surrounding crust. Diatremes with a depth (D_d) of 1000 m and with different dipping angles (α) and infill percentages (V_{infill}). Legend as in Fig. 2.

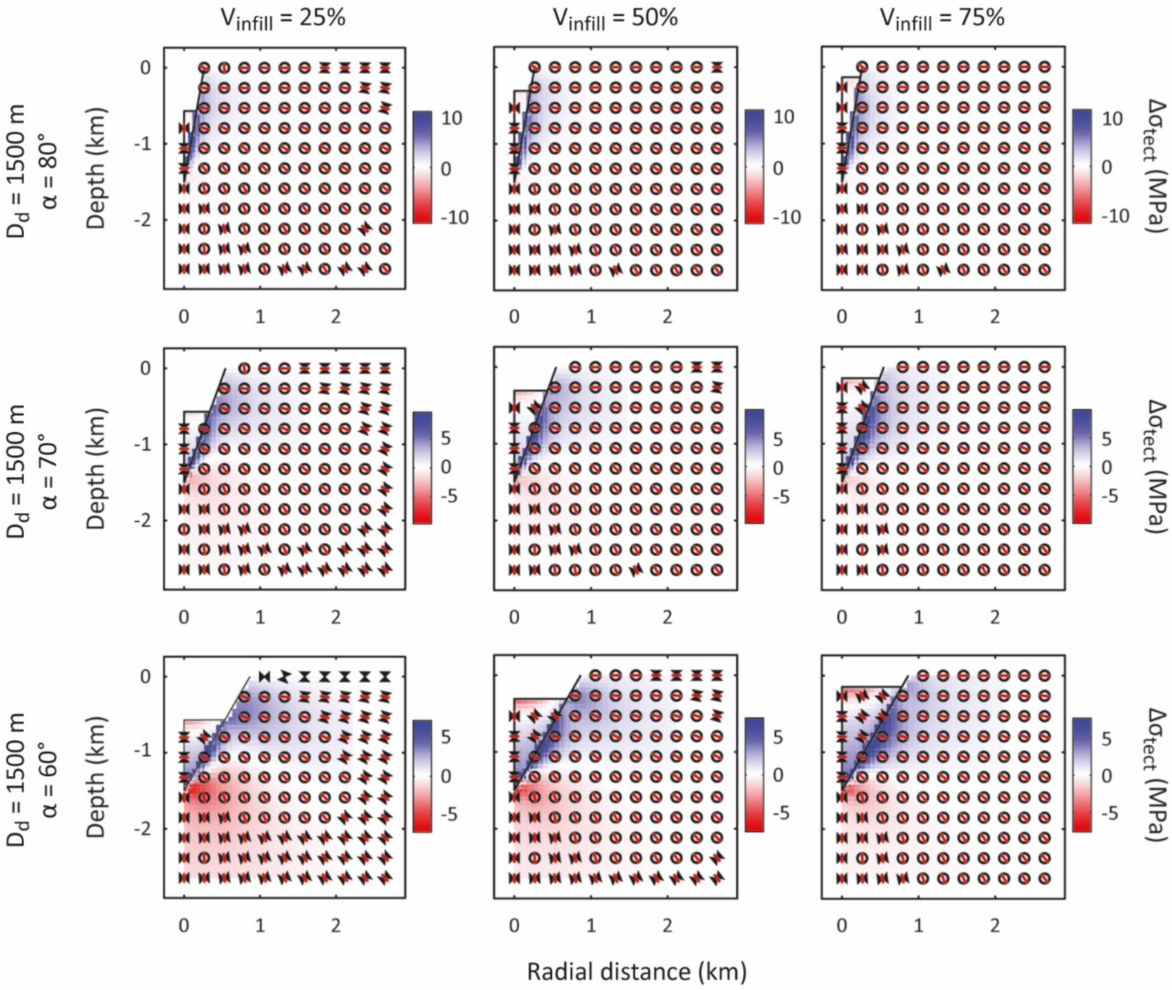

Supplementary Figure 11: State of stress within the diatreme's infill and the surrounding crust. Diatremes with a depth (D_d) of 1500 m and with different dipping angles (α) and infill percentages (V_{infill}). Legend as in Fig. 2.

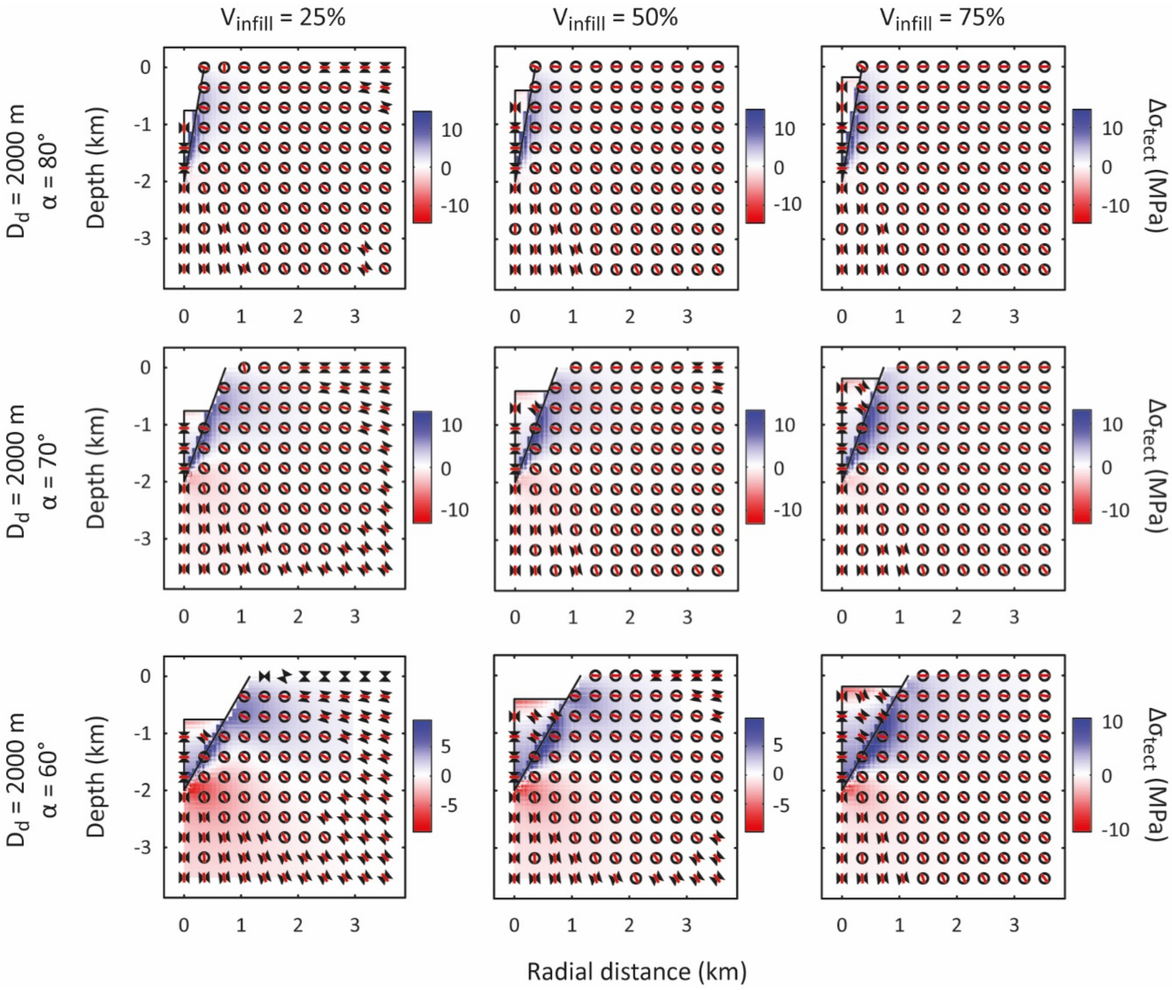

Supplementary Figure 12: State of stress within the diatreme's infill and the surrounding crust. Diatremes with a depth (D_d) of 2000 m and with different dipping angles (α) and infill percentages (V_{infill}). Legend as in Fig. 2.

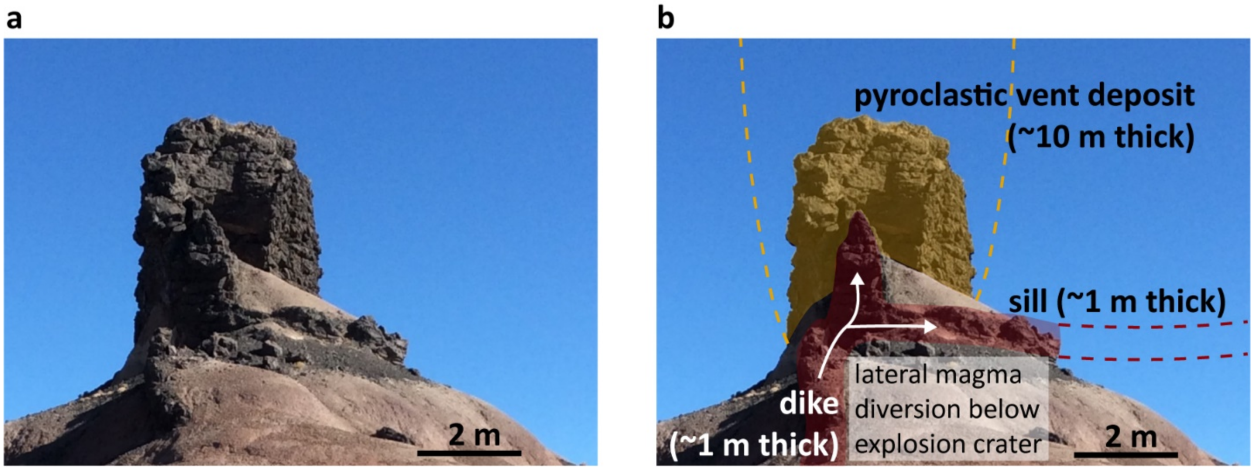

Supplementary Figure 13: Example of a dike transitioning to a sill intrusion immediately below an eruptive conduit in the Hopi Buttes volcanic field. In the photo (lat 35.10406°N, long 110.30943°W) provided (a and b), no cross-cutting relations are observed between the dike, sill, and conduit. The scale provided is in reference only to the foreground of the photo. Dashed lines in b represent an interpreted projection of the vent and sill prior to erosion. Reworked from Muirhead at al.[1].

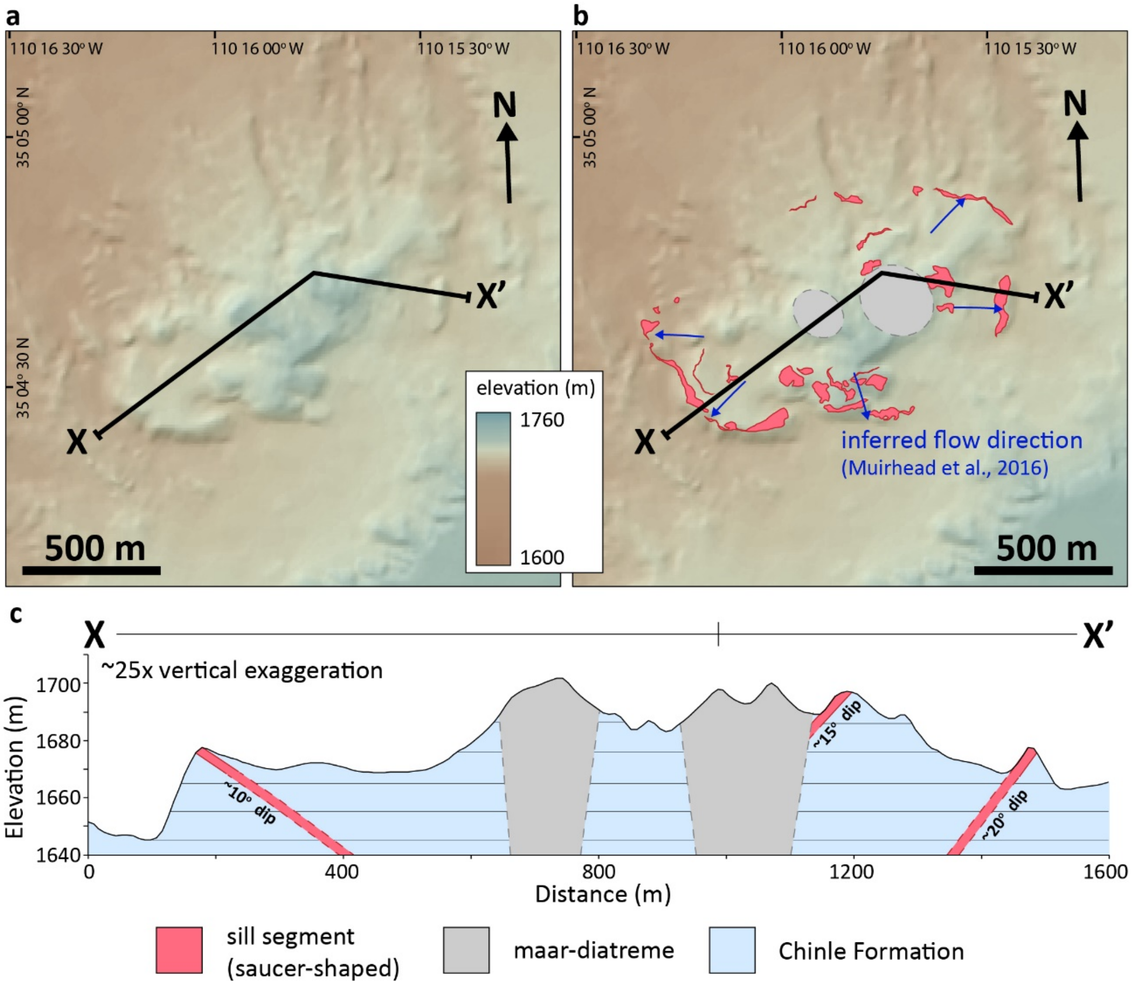

Supplementary Figure 14: Diatreme-sill complex observed at Crowne Butte in the Hopi Buttes volcanic field. a, digital elevation model of the complex. b, mapped distribution of sill segments and diatremes slightly modified from from Muirhead et al. (2016). Primary magma flow directions were based on the orientation of long-axes of sill segments observed in the field. c, geological cross-section through Crowne Butte from X-X' annotated in a and b. The thicknesses of the sill segments are exaggerated (the segments are typically ~1 m thick in the field). Intrusion dips are based on structural mapping of Muirhead et al.[1]. Overall, the aerial distribution and inward-dipping character of sill segments are consistent with a saucer-shaped morphology. Outer sills may project to the base of diatremes, where sub-vertical dikes possibly fed them.

Supplementary References

1. Muirhead JD, Van Eaton AR, Re G, White JDL, Ort MH. Monogenetic volcanoes fed by interconnected dikes and sills in the Hopi Buttes volcanic field, Navajo Nation, USA. *Bulletin of Volcanology* **78**, 11 (2016).
