# Supplementary material for: Shallow magma diversions during explosive diatreme-forming eruptions
Source: Nat Commun. 2018 Apr 13;9:1459. doi: 10.1038/s41467-018-03865-x (PMC5899137; doi:10.1038/s41467-018-03865-x)
Supplement: Supplementary file 2 — Peer Review Report [file 41467_2018_3865_MOESM2_ESM.pdf]

Reviewers' comments:

Reviewer #1 (Remarks to the Author):

Review of “Shallow magma diversions during explosive diatreme-forming eruptions”
Le Corvec, Muirhead and White

I have enjoyed reading the paper by Le Corvec et al. on the numerical modelling of volcanic diatreme formation, infilling and implications for magma intrusion geometry and propagation pathways. The paper is well-written and the results are interesting and relevant for a volcanological audience and this research deserves to be published. However, I am not sure of its broader impact and what ‘big questions’ are being addressed. This makes me question its suitability for Nature Geoscience. The modelling explores end-member scenarios; however, I am not completely convinced that substantial advancement has been made to improve our understanding of diatreme formation and impact on magma intrusions compared to what has come from existing models. I have four main concerns and then several specific points that should be addressed.

1. Impact of unloading and diatreme infill results on magma intrusions.
The modelling problem is quite similar to other crustal unloading scenarios, such as magma intrusions in a volcanic rift (e.g. Maccaferri et al. 2014) or beneath a caldera (Corbi et al. 2015; Corbi et al. 2016). The conceptual model presented by Le Corvec et al. explores the development of a diatreme that is fed by a dike breaching the surface (Fig 1a) and then filled when intrusions have been diverted due to stress evolution in the crust (Fig 4). However, the numerical model (as far as I understand) does not include a feeder dike or subsequent intrusions into the host rock. Can the authors explain what impact the presence of a feeder dike may have on the stress conditions in the unloaded crust? There are many lines of evidence which suggest that dikes ascend with a magmatic overpressure which may reach several MPa (which is comparable to some of the modelling output stress changes). The relative contributions of stress from the intrusions and the unloading/refilling should be explored or at least discussed in more detail. There will be a balance between local stresses, from the intrusions themselves and the unloading from the vent or diatreme. How important is this? How does it change over time?

2. Relevance of some parameter values
More clarity is needed in the text to explain the choice of parameter values, choice of variables and boundary conditions. The results need to be discussed more to take into account complexities such as e.g. crustal heterogeneity, or the presence of an aquifer. Some discussion is also needed to explain how relevant E, ν and ρ of the infill are and what impact this has on the model results and your conclusions. The infill will be loose, volcaniclastic sediment and in the case of kimberlite diatremes the infill may even be fluidised (e.g. Gernon et al. 2008) and so have low density, and lower than the values you have used I expect. What impact does the physical properties of the infill have on your results? You have also specified the nature of the infill contact with the crust in your model, but how relevant is this and how could it be different and affect your results?

3. Link with intra-diatreme explosions and host-rock mixing
I understand the link you have made to intrusion pathways, but am not convinced you have been able to directly link this to explosive activity or host-rock mixing (as is mentioned in the abstract).

Specific comments

Relevance of some of the supplementary materials: I think it is inappropriate to have such a large number of figures in the supplementary materials and that the paper should be able to be read and understood independently from this. However, I was interested to see the parametric study and think this does support and enhance the material presented in the main text. However, I am

not convinced that the field aspects are required in the supplementary material, as this material has been largely already published and I am not convinced we learn much more from it that couldn't be stated by referring to the published work by Muirhead et al. 2016.

Definitions: Please be clear about your definitions of magma intrusions e.g. dike, sill, cone-sheet, saucer-shaped sill, inclined sheet. These are usually defined geometrically (e.g. dip angle) but also in relation to sedimentary bedding (concordant, discordant), so please clarify how you use these terms.

Abstract:
"We demonstrate novel mechanisms explaining.."
• Rephrase, reads incomplete

"Similar mechanisms can be inferred to act at other, monitored, volcanic vents.."
• Why only monitored? Do you mean non-diatreme volcanoes, so any volcanic vent?

Introduction
• Could also include a reference to the recent paper by Castro et al. (2016) on simultaneous intrusive and extrusive activity.

Stress states affect intrusion geometries
• Lines 70 and 72: Typo, instead refer to Fig 2.1c and 2.1d

Stress states during maar-diatreme formation
• I expect that the diatreme wall angle and different depths are chosen to reflect the progressive development of the diatreme, and that these end-members represent observations of vent widening over time and vent deepening? It would be helpful if the authors could explain the reasons for their choices of variables up front. This could be done in part by moving text in lines 121-130 to earlier in the manuscript.
• Lines 94: typo fig 2a and 2c
• Lines 95: typo fig 2a and 2c
• Line 98: typo fig 2a
• Line 92-113: The results need to be explained a bit more precisely and referring more to Fig 2a and 2b in the text, e.g. '..decrease in the area..' (line 96) but for 60º angle diatreme it seems there is no region that is in horizontal extension so please state this. Figure 2a enables you to comment on the depth along the diatreme wall where extension is created and how this depth changes according to your end member scenarios, so please be more explicit and state values (min, max, average e.g. line 98). Also in these two paragraphs please be more explicit about the impact of the stress orientations, and remind the reader whether this means 'sill-forming' or 'dike-forming'. Describe in the text how in Figure 3a,b the lines overlap.
• Make sure supplementary figures are listed in order (e.g. Fig S9 is mentioned before S5, line 104-113)

Feedbacks between intrusive processes and magma fragmentation
• Line 140: I guess you mean the text and Figures S14 and S15? Please state this.

Concluding remarks
• Line 193: typo 'the crust'

Methodology
• Line 248: 'mechanical lens' – not sure what you mean here? Framework?

Figures and Tables
• Figure 2: a,b What are the different colors? Some labels seem to be missing. Add a legend

please, or add all dip values in color as is done in Figure 3a,b. Add ‘sill-forming’ or ‘dike-forming’ to different regions of graphs next to the (-) and (+) signs. Explain the position of dash line, include in the caption E, ν and ρ of the model crust.
• Figure 2 and similar: Caption: explain what the little circles mean.
• Figure 3 and similar: Include in the caption E, ν and ρ of the model crust and infill.
• Fig 3a, b, S4 and S9: yellow line and text doesn't show up well. Replace or use dash or dot to distinguish lines.
• Figure 4: Fig 4a2 the dike does not touch the base of the diatreme, is this on purpose? The schematic also suggests the sill intrusion happens as the eruption is occurring (the plume remains), so there is simultaneous intrusion and extrusion? Or is there a pause in eruption? What is the time step between Fig 4a and 4b? Is there sufficient time for stresses from the intrusions in 4a to relax? This figure needs to be explained more in the text and caption.
• Figure S14: Caption Line 119: typo ‘provided’. Is the arrow in the right place for the dike? It seems to point pointing at the horizontal sill? What is the vertical protrusion from the top of the sill? Looks like a hybrid? This seems to be modified from Muirhead et al. (2016)? Reference needed.
• Table 1 doesn't seem to be referred to in the text. In its caption explain the meaning of the symbols in the column headers.
• Fig S1: in the caption explain the meaning of Rd, Dd and state their ranges.
• Fig S2, S4, S5, S6, S7, S8: It would make more sense to order these panels by increasing dip angle as this would simulate the progressive development of the diatreme.

References

Castro, J.M. et al., 2016. Rapid laccolith intrusion driven by explosive volcanic eruption. Nature Communications, 7, pp.1–7.
Corbi, F. et al., 2015. How caldera collapse shapes the shallow emplacement and transfer of magma in active volcanoes. Earth and Planetary Science Letters, 431, pp.287–293.
Corbi, F. et al., 2016. Understanding the link between circumferential dikes and eruptive fissures around calderas based on numerical and analog models. Geophysical Research Letters.
Gernon, T.M. et al., 2008. Gas-fluidisation in an experimental tapered bed: Insights into processes in diverging volcanic conduits. Journal of Volcanology and Geothermal Research, 174(1-3), pp.49–56.
Maccaferri, F. et al., 2014. Off-rift volcanism in rift zones determined by crustal unloading. Nature Geoscience, 7(4), pp.297–300.
Muirhead, J.D. et al., 2016. Monogenetic volcanoes fed by interconnected dikes and sills in the Hopi Buttes volcanic field, Navajo Nation, USA. Bulletin of volcanology, 78(2), pp.93–16.

Reviewer #2 (Remarks to the Author):

The authors present results of numerical modelling for the stress state found in the surrounding crustal rocks and within the pipe infill. Their modelling uses the package COMSOL and is set up to show the stress magnitudes and orientations during pipe excavation and during infilling of the pipe. They claim that the results of their modelling can be used to explain whether magma rises as dykes through the conduit or stalls at depths below the conduit or within the pipe (to form sills). Pipe infilling is is shown to cause rotations in the stress fields to allow renewed ascent within the pipe or to feed phreatomagmatic events within the filled conduit.

Although the results are not particularly novel they are interesting and offer a viable explanation for an array of observations from maar volcanoes. The results complement the existing literature and will be of interest to volcanologists working on mafic explosive systems.

The work is solid and the results seem internally consistent. It is impossible to evaluate the

numerical results in detail without duplicating the COMSOL application, however, the results (fig. 2 & 3) seem valid. I think the results will be of use to the field of volcanology although not transformative in nature.

Detailed Suggestions for Improvement:

1) Lines 9-13. I found this sentence very long and ineffective because it contains too many disparate thoughts.
2) Line 13. Use of the word “innovative”. I did not see anything particularly innovative about the modelling. If it is innovative, then, somewhere in the text you need to show the innovation (rather than just claim it).
3) Line 22. Use of the word “novel”. What are these novel mechanisms. Again if you are going to use this word to sell your work, then, you need to explain the novelty somewhere in the text.
4) Line 28. “Hazardous” - Why are you asserting that vent-site shifts are hazardous? Is this germane at all to the thesis of the manuscript. “Hazards” do not seem to be relevant to the rest of the manuscript. So I would stay on topic.
5) Line 33. “where” I would re-write the sentence to make it more clear that you are offering an alternate explanation to conventional hypotheses.
6) Line 36-45. I did not find this paragraph as needed to your thesis. It seems me as a weak attempt to broaden the impact of the paper; instead it detracts from the thesis and uses up words that could be used later to explain your results better.
7) Line 67. Is tectonic stress the correct or best word for the stress field you are calculating? Is it not simpler to simply refer to stresses in the stress field in and around the pipe? I have not expertise in this, but tectonic stresses have a different connotation for me.
Also, you should define sigma r and sigma z.
8) line 98-99. Are there consequences to compressive stresses of 80 MPa - those seem to me to be very high differential stresses in the shallow crust.
9) line 100. Please rewrite for clarity the phrase “in the modeled r-z plane”. Perhaps you are just missing commas but I found the sentence ambiguous.
10) line 102. “Directly below the surface” - is this referring to the Earth’s surface or another surface?
11) Last comment. Figures 2 and 3 are central to the papers ideas as they are the results of the modelling. Are there ways to make them clearer or more accessible. They are used to illustrate alot of concepts referred to in the text but I found them alot of work to unravel and not particularly useful. Is there a way to colour better or contour the results. If that is the standard in the field then perhaps they are good enough and the informed will have no trouble accessing the concepts.

In summary, the authors use numerical modelling to track an evolving stress field attending pipe excavation and subsequent infilling during explosive (maar-style) eruption. Their results are used to explain previously documented field relationships found in maar style volcanoes and explain dyke vs sill injections, as well as, the variable locations (within pipe) of phreatomagmatic explosive events. The results will have impact on the volcanological community and contribute to a growing literature on magma migration.

Response to Reviewers' comments:

Reviewer #1 (Remarks to the Author):

Review of "Shallow magma diversions during explosive diatreme-forming eruptions" Le Corvec, Muirhead and White

I have enjoyed reading the paper by Le Corvec et al. on the numerical modelling of volcanic diatreme formation, infilling and implications for magma intrusion geometry and propagation pathways. The paper is well-written and the results are interesting and relevant for a volcanological audience and this research deserves to be published. However, I am not sure of its broader impact and what 'big questions' are being addressed. This makes me question its suitability for Nature Geoscience. The modelling explores end-member scenarios; however, I am not completely convinced that substantial advancement has been made to improve our understanding of diatreme formation and impact on magma intrusions compared to what has come from existing models. I have four main concerns and then several specific points that should be addressed.

General Response: *[line numbers given match revised text with markup hidden/removed***]**

We are pleased to hear that the reviewer enjoyed reading the paper and finds it well-written, interesting and relevant. Importantly, we would like to point out that this is the *first study to model stress fields related to diatreme formation*. The stress changes and two-step model of excavation and infilling characteristic of explosive eruptions has never been modeled before, yet represents a process common to all explosive eruptions (see Lines 8-13). This study thus represents a significant advance in our understanding of how diatreme formation and explosive volcanism can effect stress fields and intrusion propagation.

We have made changes to the text, as detailed below, to better communicate the substantial advance made in our work.

1. Impact of unloading and diatreme infill results on magma intrusions.

The modelling problem is quite similar to other crustal unloading scenarios, such as magma intrusions in a volcanic rift (e.g. Maccaferri et al. 2014) or beneath a caldera (Corbi et al. 2015; Corbi et al. 2016). The conceptual model presented by Le Corvec et al. explores the development of a diatreme that is fed by a dike breaching the surface (Fig 1a) and then filled when intrusions have been diverted due to stress evolution in the crust (Fig 4). However, the numerical model (as far as I understand) does not include a feeder dike or subsequent intrusions into the host rock. Can the authors explain what impact the presence of a feeder dike may have on the stress conditions in the unloaded crust? There are many lines of evidence

which suggest that dikes ascend with a magmatic overpressure which may reach several MPa (which is comparable to some of the modelling output stress changes). The relative contributions of stress from the intrusions and the unloading/refilling should be explored or at least discussed in more detail. There will be a balance between local stresses, from the intrusions themselves and the unloading from the vent or diatreme. How important is this? How does it change over time?

Finite element modeling (FEM) is a common tool for testing mechanical processes of magma propagation through the crust [*Maccaferri et al.*, 2014; *Corbi et al.*, 2015; *Corbi et al.*, 2016]. Our approach is different, however, from the finite element models cited by the reviewers, which involve calculation of only a one-step process in 2D (aka plane strain). In contrast, our work computes in 2D axisymmetry (i.e., 3D) a two-step process involving 1st excavation of a gravitationally loaded lithosphere, and 2nd the filling of that newly created cavity while retaining the initial stress response of the excavation process.

The FEMs that can model dike propagation only exist in 2D, and therefore lack 3D information about the state of stress. Some studies referred to by the reviewer above encountered the same limitation for their numerical simulations (i.e., [*Corbi et al.*, 2015; *Corbi et al.*, 2016]), but instead infer the likely orientation of intrusions based on the 3D stress field due to unloading processes, which is corroborated by combined numerical and analogue studies [*Corbi et al.*, 2016]. ***Discussion on these points are now added to Lines 241-244.***

We agree with this reviewer that an intrusion generates its own stress field that will interact with the local state of stress. This is not only challenging to model in a static 3D stress field (see above), but will also vary dynamically. Specific levels of magma pressure and thus of the amount of stress generated are a matter of debate. Theoretical work shows that the propagating tip of a dike with basaltic magma is generally led by a buoyant head [*Menand and Tait*, 2002; *Taisne and Tait*, 2009; *Geshi et al.*, 2010], where the overpressure is localized. We agree with the reviewer that the amount of overpressure within the dike can reach several MPa (Lines 143-144), yet this stress will vary temporally and spatially, particularly during explosive volcanic events like those we are testing. ***For example, the overpressure at the head of the dike will be released during explosive fragmentation in stage 1. The magma subsequently delivered from the dike's tail carries much lower overpressure than the head (<< MPa) [Lister and Kerr, 1991]*** (Lines 144-146). Despite these complications, the influence of the stresses generated by either the excavation process or the infill process is much greater than that theoretically expected from the dike, and thus intrusions paths are expected to closely mimic the modelled stress field (i.e., [*Corbi et al.*, 2015; *Corbi et al.*, 2016]).

Modeling successive intrusions is beyond the scope of this paper. Looking at maar-diatreme eruptions, we consider the system to be monogenetic, with only one period of magma delivery sustained for hours to years. ***The goal of this paper is not to present a full dynamic model, but to demonstrate that there are clear stress effects that result from diatreme unloading/loading, and illustrate areas prone to sill vs dike intrusion.***

2. Relevance of some parameter values

More clarity is needed in the text to explain the choice of parameter values, choice of variables and boundary conditions. The results need to be discussed more to take into account complexities such as e.g. crustal heterogeneity, or the presence of an aquifer. Some discussion is also needed to explain how relevant E, ν and ρ of the infill are and what impact this has on the model results and your conclusions. The infill will be loose, volcaniclastic sediment and in the case of kimberlite diatremes the infill may even be fluidised (e.g. Gernon et al. 2008) and so have low density, and lower than the values you have used I expect. What impact does the physical properties of the infill have on your results? You have also specified the nature of the infill contact with the crust in your model, but how relevant is this and how could it be different and affect your results?

In line with the reviewer's comments, **we have performed sensitivity tests** for different infill properties, and these are discussed and presented the Supplementary Material. ***In the main text (Line 176-178) we note that "For a range of plausible infill properties (sensitivity tests in Supplementary Material), horizontal extension…"***

No published work provides data on physical characteristics of unlithified diatreme infill, so we consider a range of values between a loose granular material and a weak brittle rock. [*Woolsey et al.*, 1975; *Gernon et al.*, 2009] infer zones of host rock fluidization during eruption of kimberlite diatremes, but not within the static deposits into which a dike would intrude (Line 261-262). Magmatic intrusions observed in the field show that the infill allows both propagation of magma-filled cracks, and physical mingling of infill with magma. This implies different strain rates during dike propagation, changes through time in infill properties, spatial variability of infill properties, or most probably, all of the above.

Results of the sensitivity test show that the stress orientations remain the same for the full range of elastic parameters, with some changes in the magnitudes of these stresses for different Young's Modulus values. The same intrusion orientations are expected regardless of the elastic properties of the infill, and the range of stress magnitudes are still high enough to generate the magma diversions as discussed. Based on these sensitivity tests, ***we now present a more conservative value of Young's Modulus (2.5 GPa) for the***

figures in the main text and in Supplemental Material. The stress fields, however, remain unchanged, as have our interpretation, discussion, and conclusions (see Supplemental Material).

Simplification of a natural process is the key to modelling it. Here we focus specifically on the mechanical role of decompression resulting from diatreme excavation, and on partial recompression during its subsequent filling. The model does address the key crustal heterogeneity relevant to the scale and scope of the current study, by modelling crater exactions and diatreme infilling in an elastic host. Adding the most probable crustal heterogeneity, which is layers with different properties in the elastic host, would not affect our conclusions. Layering is often not included in elastic modeling studies like ours testing first-order stress effects. However, as shown in previous studies (e.g., [*Brenner and Gudmundsson*, 2004]) and ***discussed in the text (line 91-92)***, and *layering will only enhance the stress field rotations* discussed and presented, further promoting sill formation below the diatreme.

Our model does not address complexities of local hydrology or effects of water on the explosions in order to, as in many other similar finite element modelling studies [*Maccaferri et al.*, 2014; *Corbi et al.*, 2015; *Maccaferri et al.*, 2015; *Corbi et al.*, 2016; *Pinel et al.*, 2017], focus on the unloading/loading processes. However, effects of water on properties of the diatreme infill, such as cohesion, are captured broadly in the range of Young's Moduli now considered in the sensitivity tests.

Fracturing would dissipate part of the accumulated stress within the country rock, but the magnitudes (tens of MPa) reached during excavation are sufficiently large, compared to the amount of stress released by brittle rock failure, to influence the propagation and the direction of propagation of the magma intrusion [*Kaiser and Tang*, 1998]. In addition, the amount of stress released is highly dependent of the initial conditions of the material (e.g., pre-existing fractures). ***We have added description of such interactions in the main text (Line 163-166).***

Finally, the infill contact is free to move along the diatreme wall which allows decoupling between the infill domain and the crustal domain. Having a coupled contact would be unrealistic for loose debris resting against an inclined wall. Line 271-273.

The limitations arising from poor constraints for some model parameters result in unavoidable uncertainties in our simulations, and thus we address these through the newly presented sensitivity test. A future way to address uncertainties is to make use of a probabilistic approach. For instance, assigning likelihood distributions to the input parameters, running the model several thousand times, and then computing the probability to get a certain result (i.e., a “family” of dyke propagation paths). Such stochastic approaches are computationally expensive and would require further optimization of our code

and parallelization of the model runs. This paper provides the basis on which to build a project of this sort in the future.

3. Link with intra-diatreme explosions and host-rock mixing

I understand the link you have made to intrusion pathways, but am not convinced you have been able to directly link this to explosive activity or host-rock mixing (as is mentioned in the abstract).

We agree that the link between intrusion pathways and explosive activity within the diatreme is not modelled in this study. The novelty and main conclusions focus on stress interactions during diatreme formation, and discussion of explosive fragmentation has been included to place these results within a broader context. Explosive fragmentation in our study is inferred to be favored where the modelled stresses favor stalling of magma at shallow depths in the diatreme and lateral spreading of magma as a sill. Spreading of magma in the diatreme fill increases the probability of magma interacting with available groundwater to drive explosive phreatomagmatic eruptions. Revised conceptual models of diatreme formation (Valentine and White, 2012) suggest a preference for explosive fragmentation at shallow depths over a larger area to explain upward diatreme widening. Valentine and White (2012) hypothesize that “intra-diatreme dikes are expected to follow irregular paths rather than simple vertical paths as they extend upward” – our numerical model results provide a mechanism driving these “irregular” intrusion pathways that allow for more efficient host rock mixing at shallow depths.

We have modified the text to better emphasize this, as listed below.

Lines 135-140: “The progressive up-structure widening of the diatreme in the latter models results from collapse of unsupported wallrock [*White and Ross*, 2011], explosive fragmentation at shallower depths [*Sweeney and Valentine*, 2015], and also **from lateral magma diversions, possibly in sills in the upper diatreme [*Valentine and van Wyk de Vries*, 2014]**. "Such models thus **require that vertically ascending magma preferentially stalls in the shallow subsurface, which our model shows is favored by the stress response to infilling."**

Lines 179-181: “As predicted by Valentine and White (2012), **our results show that the orientation and magnitude of σ_3 within the infill can also promote irregular magma propagation paths** (sub-vertical to inclined), and that magma can be drawn to the peripheries of the diatreme.”

Lines 184-190; "…stalling ascending magma and promoting **magma-host mingling or** lateral diversion…", and magma "…will enter an area of horizontal extension **that favors groundwater entry and thus phreatomagmatic explosions…**"

Specific comments

Relevance of some of the supplementary materials: I think it is inappropriate to have such a large number of figures in the supplementary materials and that the paper should be able to be read and understood independently from this. However, I was interested to see the parametric study and think this does support and enhance the material presented in the main text. However, I am not convinced that the field aspects are required in the supplementary material, as this material has been largely already published and I am not convinced we learn much more from it that couldn't be stated by referring to the published work by Muirhead et al. 2016.

We have reduced the number of the original figures in the Supplementary Material. We have focus the parametric study on one case, i.e., the constant depth case. We have, however, included some new figures in response to the sensitivity test requested by the reviewer. The total number of figures presented in the supplemental text is appropriate for a comprehensive modeling studying testing various parameter space. The cross-section of Crown Butte was not published in Muirhead et al. (2016), and thus makes an important contribution this paper, which the reader can compare with model predictions. We therefore believe it should be included.

Definitions: Please be clear about your definitions of magma intrusions e.g. dike, sill, cone-sheet, saucer-shaped sill, inclined sheet. These are usually defined geometrically (e.g. dip angle) but also in relation to sedimentary bedding (concordant, discordant), so please clarify how you use these terms.

The term cone-sheet has been removed from the paper. In line with previous studies (e.g., Muirhead et al., 2016), we now define intrusions based on the dip angle. For the term saucer-shaped sill the reader is referred to Muirhead et al. (2016) and references therein.

Abstract:

“We demonstrate novel mechanisms explaining..”

• Rephrase, reads incomplete

Sentence reworded.

“Similar mechanisms can be inferred to act at other, monitored, volcanic vents..”

• Why only monitored? Do you mean non-diatreme volcanoes, so any volcanic vent?

Changed to: “Similar mechanisms can be inferred from monitoring data to act at other volcanic vents..”

Introduction

• Could also include a reference to the recent paper by Castro et al. (2016) on simultaneous intrusive and extrusive activity.

Added to the manuscript.

Stress states affect intrusion geometries

• Lines 70 and 72: Typo, instead refer to Fig 2.1c and 2.1d

We have changed the figure callout from “Fig. 2.1” to “Fig. 2.1c” in Lines 70 and 73 and since both states of stress are observable in Fig. 2.1c (i.e., compressional and extensional, in red and blue, respectively).

Stress states during maar-diatreme formation

• I expect that the diatreme wall angle and different depths are chosen to reflect the progressive development of the diatreme, and that these end-members represent observations of vent widening over time and vent deepening? It would be helpful if the authors could explain the reasons for their choices of variables up front. This could be done in part by moving text in lines 121-130 to earlier in the manuscript.

The geometrical parametric analysis represents different sizes corresponding with the natural sizes observed in nature. We have added references in the methodology to support our choices.

• Lines 94: typo fig 2a and 2c

Corrected.

• Lines 95: typo fig 2a and 2c

Corrected.

• Line 98: typo fig 2a

Corrected.

• Line 92-113: The results need to be explained a bit more precisely and referring more to Fig 2a and 2b in the text, e.g. ’..decrease in the area..’ (line 96) but for 60º angle diatreme it seems there is no region that is in horizontal extension so please state this. Figure 2a enables you to comment on the depth along the diatreme wall where extension is created and how this depth changes according to your end member scenarios, so please be more explicit and state values (min, max, average e.g. line 98). Also in these two paragraphs please be more explicit about the impact of the stress orientations, and remind the reader whether this means ‘sill-forming’ or ‘dike-forming’. Describe in the text how in Figure 3a,b the lines overlap.

We changed the text to be more specific. We quantify the area subjected to extensional and compressional stresses on the diatreme walls.

Maximum stress values and orientations are stated in the critical zones we discuss in the discussion section of the paper.

We have also added more explicitly maximum and minimum for the values used in the text.

As suggested, we now explicitly discuss the type of intrusion favored by the stress orientation in each result section.

• Make sure supplementary figures are listed in order (e.g. Fig S9 is mentioned before S5, line 104-113)

We have updated the order of the figures. We have also decreased the number of figures in the Supplementary Material based on the comments of the other reviewer.

Feedbacks between intrusive processes and magma fragmentation

• Line 140: I guess you mean the text and Figures S14 and S15? Please state this.

Done.

Concluding remarks

• Line 193: typo 'the crust'

Corrected.

Methodology

• Line 248: 'mechanical lens' – not sure what you mean here? Framework?

Sentence modified. "…modelling show the potential for country-rock responses to modulate magma behavior during evolution of diatremes…" (Lines 277-278).

Figures and Tables

• Figure 2: a,b What are the different colors? Some labels seem to be missing. Add a legend please, or add all dip values in color as is done in Figure 3a,b. Add 'sill-forming' or 'dike-forming' to different regions of graphs next to the (-) and (+) signs. Explain the position of dash line, include in the caption E, ν and ρ of the model crust.

We have added color descriptions to the caption, and labels in Fig 2a and 2b. We have added an axis close to Fig. 2c to show the r-z plane. As we cannot model intrusion propagation into the 3D stress fields, it

would be inaccurate for us to include ‘sill-forming’ vs. ‘dike-forming’ regions of the plots. Our study shows areas that are prone to sill or dike intrusion, but the final intrusion geometry is still dependent on the dynamic stress interactions discussed for General Comment 1. The position of the dash line at 0 MPa is the limit between the positive (+) and negative (-) values, i.e. between the areas under horizontal extensional and compressional stresses, also represented in blue and red shading in Fig. 2c-d (cf. Lines 69-73).

• Figure 2 and similar: Caption: explain what the little circles mean.

“Circles represent end-on hourglass shapes (σ_1 perpendicular to the r-z plane).”

• Figure 3 and similar: Include in the caption E, ν and ρ of the model crust and infill.

We have added the values in the caption of Figure 1.

• Fig 3a, b, S4 and S9: yellow line and text doesn't show up well. Replace or use dash or dot to distinguish lines.

Done.

• Figure 4: Fig 4a2 the dike does not touch the base of the diatreme, is this on purpose? The schematic also suggests the sill intrusion happens as the eruption is occurring (the plume remains), so there is simultaneous intrusion and extrusion? Or is there a pause in eruption? What is the time step between Fig 4a and 4b? Is there sufficient time for stresses from the intrusions in 4a to relax? This figure needs to be explained more in the text and caption.

The gap between the base of the diatreme and the dike in Fig. 4a2 has been decreased. Reorientation of magma intrusions could occur within the crust below the base of the diatreme is shown in Fig. 2a and b, where the amount of horizontal compressional stress reaches values exceeding several MPa at several hundreds of meters or kilometers depth, depending of the size of the initial crater. The plume in Fig. 4a2 is somewhat diagrammatic, but suspended ash remains in the atmosphere during short gaps between explosions, and there are also ashy gash and steam emissions, so a completely clear-sky crater is unlikely during an eruption.

• Figure S14: Caption Line 119: typo ‘provided’. Is the arrow in the right place for the dike? It seems to point pointing at the horizontal sill? What is the vertical protrusion from the top of the sill? Looks like a hybrid? This seems to be modified from Muirhead et al. (2016)? Reference needed.

Typo corrected. Figure modified to address comments. Reference added.

• Table 1 doesn't seem to be referred to in the text. In its caption explain the meaning of the symbols in the column headers.

We now refer to Table S1 on Line 255 in the Methodology section.

• Fig S1: in the caption explain the meaning of Rd, Dd and state their ranges.

Done.

• Fig S2, S4, S5, S6, S7, S8: It would make more sense to order these panels by increasing dip angle as this would simulate the progressive development of the diatreme.

We displayed the figures based on constant radius with different dip angle of the diatreme's flank. We have tested different options and this one was the simplest one to avoid excessive mixing between the figures.

References

Castro, J.M. et al., 2016. Rapid laccolith intrusion driven by explosive volcanic eruption. Nature Communications, 7, pp.1–7.

Corbi, F. et al., 2015. How caldera collapse shapes the shallow emplacement and transfer of magma in active volcanoes. Earth and Planetary Science Letters, 431, pp.287–293.

Corbi, F. et al., 2016. Understanding the link between circumferential dikes and eruptive fissures around calderas based on numerical and analog models. Geophysical Research Letters.

Gernon, T.M. et al., 2008. Gas-fluidisation in an experimental tapered bed: Insights into processes in diverging volcanic conduits. Journal of Volcanology and Geothermal Research, 174(1-3), pp.49–56.

Maccaferri, F. et al., 2014. Off-rift volcanism in rift zones determined by crustal unloading. Nature Geoscience, 7(4), pp.297–300.

Muirhead, J.D. et al., 2016. Monogenetic volcanoes fed by interconnected dikes and sills in the Hopi Buttes volcanic field, Navajo Nation, USA. Bulletin of volcanology, 78(2), pp.93–16.

<u>Reviewer #2 (Remarks to the Author):</u>

The authors present results of numerical modelling for the stress state found in the surrounding crustal rocks and within the pipe infill. Their modelling uses the package COMSOL and is set up to show the stress magnitudes and orientations during pipe excavation and during infilling of the pipe. They claim that

the results of their modelling can be used to explain whether magma rises as dykes through the conduit or stalls at depths below the conduit or within the pipe (to form sills). Pipe infilling is is shown to cause rotations in the stress fields to allow renewed ascent within the pipe or to feed phreatomagmatic events within the filled conduit.

Although the results are not particularly novel they are interesting and offer a viable explanation for an array of observations from maar volcanoes. The results complement the existing literature and will be of interest to volcanologists working on mafic explosive systems.

The work is solid and the results seem internally consistent. It is impossible to evaluate the numerical results in detail without duplicating the COMSOL application, however, the results (fig. 2 & 3) seem valid. I think the results will be of use to the field of volcanology although not transformative in nature.

As now clarified further in the revised text and discussed in previous response, we believe this work is novel and broadly applicable to all explosive eruptions (see Lines 8-13). It is the first study to model stress fields related to diatreme formation, and provides new mechanism for producing complex intrusion geometries new explosive vents, as well as feedbacks between explosive cratering and intrusion processes.

Detailed Suggestions for Improvement:

1) Lines 9-13. I found this sentence very long and ineffective because it contains too many disparate thoughts.

Rewritten to improve clarity.

2) Line 13. Use of the word “innovative”. I did not see anything particularly innovative about the modelling. If it is innovative, then, somewhere in the text you need to show the innovation (rather than just claim it).

The two-step model of excavation and infilling characteristic of explosive eruptions has never been modeled before. We now describe in the text why our work is innovative:

Lines 43-45: “Here we utilize, for the first time, finite element modeling to analyze the evolution of stress states during explosive excavation and filling of gravitationally loaded country rock.”

Line 235-237: “We use finite element models through COMSOL Multiphysics® to investigate for the first time the effects of diatreme excavation for single-explosion craters followed by the infilling of the newly formed diatreme on the local state of stress”

3) Line 22. Use of the word “novel”. What are these novel mechanisms. Again if you are going to use this word to sell your work, then, you need to explain the novelty somewhere in the text.

See comment above.

4) Line 28. “Hazardous” - Why are you asserting that vent-site shifts are hazardous? Is this germane at all to the thesis of the manuscript. “Hazards” do not seem to be relevant to the rest of the manuscript. So I would stay on topic.

Volcanic eruptions are hazardous by nature in populated regions, particularly when there are sudden vent shifts. Understanding what makes magma divert laterally thus has broad implications for volcanic hazards, and thus it is important to mention this to provide a broader context for the study. We have now adjusted the introduction to emphasize this point early in the paper:

Line 32-35: “The feedbacks between magma plumbing system development and the location of magmatic fragmentation need to be well-understood to forecast eruptions and mitigate risk, particularly in densely populated areas situated on volcanic fields (e.g., Mexico City, Mexico, and Auckland, New Zealand).”

5) Line 33. “where” I would re-write the sentence to make it more clear that you are offering an alternate explanation to conventional hypotheses.

Done.

6) Line 36-45. I did not find this paragraph as needed to your thesis. It seems me as a weak attempt to broaden the impact of the paper; instead it detracts from the thesis and uses up words that could be used later to explain your results better.

Agreed. We’ve removed this paragraph.

7) Line 67. Is tectonic stress the correct or best word for the stress field you are calculating? Is it not simpler to simply refer to stresses in the stress field in and around the pipe? I have not expertise in this, but tectonic stresses have a different connotation for me.

We use an existing terminology (e.g., [*Galgana et al.*, 2011; *Galgana et al.*, 2013; *Le Corvec et al.*, 2015] and reference therein). The term tectonic here is used to distinguish the differential tectonic stress from the differential stress (sigma 1 – sigma 3) which corresponds only to the magnitude of the stress field within the lithosphere. The differential tectonic stress however consider the relative signs of the principal stresses, as explained in the beginning of the section “stress states affect intrusion geometries”.

Also, you should define sigma r and sigma z.

Sigma r and sigma z are just the stress within the radial (horizontal) and vertical direction. We have defined their meaning in the manuscript. Line 67 – 68.

8) line 98-99. Are there consequences to compressive stresses of 80 MPa - those seem to me to be very high differential stresses in the shallow crust.

As occurs in all elastic models like these [*Grosfils*, 2007; *Hurwitz et al.*, 2009; *Galgana et al.*, 2011; *Galgana et al.*, 2012; *Grosfils et al.*, 2013; *Maccaferri et al.*, 2014; *Le Corvec et al.*, 2015; *Maccaferri et al.*, 2015; *Maccaferri et al.*, 2017], the amount of horizontal tectonic stress reaches very high values. The explosion would generate a dense fracture network surrounding the diatreme which may cause to the medium to response in an inelastic manner. The damage zone would decrease the magnitude of the stress within the medium but would not change the main characteristics of the horizontal and extensional stress.

9) line 100. Please rewrite for clarity the phrase “in the modeled r-z plane”. Perhaps you are just missing commas but I found the sentence ambiguous.

We have added a coordinate system in the figure to show the r-z plane. Since we are modeling an axisymmetric system, we are representing only a slice of the entire computed model. Text changed (see next comment).

10) line 102. “Directly below the surface” - is this referring to the Earth’s surface or another surface?

Yes – sentence reworded to improve clarity. **Line 105-110: "The orientation of σ_3 is vertical below the excavated diatreme, favoring sill formation, but rotates in the illustrated vertical r-z plane of the model in an anticlockwise manner to become sub-horizontal in the surrounding crust, which favors dike formation (red lines Figs. 2c-d and S2). Finally, σ_3 rotates further, immediately below the ground surface, into an orientation out of the illustrated plane (hourglasses without red lines, Figs. 2c-d and S2)."**

11) Last comment. Figures 2 and 3 are central to the papers ideas as they are the results of the modelling. Are there ways to make them clearer or more accessible. They are used to illustrate alot of concepts referred to in the text but I found them alot of work to unravel and not particularly useful. Is there a way to colour better or contour the results. If that is the standard in the field then perhaps they are good enough and the informed will have no trouble accessing the concepts.

We agree that the figures are essential aids to understanding the nature of our models. This type of figure is quite commonly used to illustrate finite element modelling results [*Grosfils et al.*, 2013; *Maccaferri et al.*, 2014; *Corbi et al.*, 2015; *Corbi et al.*, 2016; *Maccaferri et al.*, 2017]. Within the plots *only two*

variables are presented, stress orientation and magnitude, which are central to the discussion of the paper, and need to be presented together to accurately interpret results.

Contouring the results, e.g. for stress alone, or stress orientation alone, would further increase the number of diagrams required, and could be individually misleading We have retained the basic format for these diagrams, and note that figure 4 is meant to present an accessible summary of our key results.

In summary, the authors use numerical modelling to track an evolving stress field attending pipe excavation and subsequent infilling during explosive (maar-style) eruption. Their results are used to explain previously documented field relationships found in maar style volcanoes and explain dyke vs sill injections, as well as, the variable locations (within pipe) of phreatomagmatic explosive events. The results will have impact on the volcanological community and contribute to a growing literature on magma migration.

References

Brenner, S. L., and A. Gudmundsson (2004), Arrest and aperture variation of hydrofractures in layered reservoirs, *Geological Society, London, Special Publications*, *231*(1), 117-128, doi:10.1144/gsl.sp.2004.231.01.08.

Corbi, F., E. Rivalta, V. Pinel, F. Maccaferri, and V. Acocella (2016), Understanding the link between circumferential dikes and eruptive fissures around calderas based on numerical and analog models, *Geophysical Research Letters*, *43*(12), 6212-6219, doi:10.1002/2016GL068721.

Corbi, F., E. Rivalta, V. Pinel, F. Maccaferri, M. Bagnardi, and V. Acocella (2015), How caldera collapse shapes the shallow emplacement and transfer of magma in active volcanoes, *Earth and Planetary Science Letters*, *431*, 287-293, doi:http://dx.doi.org/10.1016/j.epsl.2015.09.028.

Galgana, G., P. J. McGovern, and E. B. Grosfils (2012), The formation of giant radiating dike systems on Venus: Insights from elastoplastic flexural models, in *LPSC 43rd*, edited, Woodlands, Texas.

Galgana, G. A., E. B. Grosfils, and P. J. McGovern (2013), Radial dike formation on Venus: Insights from models of uplift, flexure and magmatism, *Icarus*, *225*(1), 538-547, doi:http://dx.doi.org/10.1016/j.icarus.2013.04.020.

Galgana, G. A., P. J. McGovern, and E. B. Grosfils (2011), Evolution of large Venusian volcanoes: Insights from coupled models of lithospheric flexure and magma reservoir pressurization, *J. Geophys. Res.*, *116*(E3), E03009, doi:10.1029/2010je003654.

Gernon, T. M., M. A. Gilbertson, R. S. J. Sparks, and M. Field (2009), The role of gas-fluidisation in the formation of massive volcaniclastic kimberlite, *Lithos*, *112*, 439-451, doi:https://doi.org/10.1016/j.lithos.2009.04.011.

Geshi, N., S. Kusumoto, and A. Gudmundsson (2010), Geometric difference between non-feeder and feeder dikes, *Geology*, *38*(3), 195-198, doi:10.1130/g30350.1.

Grosfils, E. B. (2007), Magma reservoir failure on the terrestrial planets: Assessing the importance of gravitational loading in simple elastic models, *Journal of Volcanology and Geothermal Research*, *166*(2), 47-75, doi:10.1016/j.jvolgeores.2007.06.007.

Grosfils, E. B., P. J. McGovern, P. M. Gregg, G. A. Galgana, D. M. Hurwitz, S. M. Long, and S. R. Chestler (2013), Elastic models of magma reservoir mechanics: a key tool for investigating planetary volcanism, *Geological Society, London, Special Publications*, *401*, doi:10.1144/sp401.2.

Hurwitz, D. M., S. M. Long, and E. B. Grosfils (2009), The characteristics of magma reservoir failure beneath a volcanic edifice, *Journal of Volcanology and Geothermal Research*, *188*(4), 379-394, doi:http://dx.doi.org/10.1016/j.jvolgeores.2009.10.004.

Kaiser, P. K., and C. A. Tang (1998), Numerical Simulation of Damage Accumulation and Seismic Energy Release During Brittle Rock Failure—Part II: Rib Pillar Collapse, *International Journal of Rock Mechanics and Mining Sciences*, *35*(2), 123-134, doi:https://doi.org/10.1016/S0148-9062(97)00010-7.

Le Corvec, N., P. J. McGovern, E. B. Grosfils, and G. Galgana (2015), Effects of crustal-scale mechanical layering on magma chamber failure and magma propagation within the Venusian lithosphere, *Journal of Geophysical Research: Planets*, *120*(7), 2015JE004814, doi:10.1002/2015je004814.

Lister, J. R., and R. C. Kerr (1991), Fluid-mechanical models of crack propagation and their application to magma transport in dykes, *Journal of Geophysical Research*, *96*(B6), 10,049-010,077.

Maccaferri, F., V. Acocella, and E. Rivalta (2015), How the differential load induced by normal fault scarps controls the distribution of monogenic volcanism, *Geophysical Research Letters*, *42*(18), 7507-7512, doi:10.1002/2015GL065638.

Maccaferri, F., N. Richter, and T. R. Walter (2017), The effect of giant lateral collapses on magma pathways and the location of volcanism, *Nature Communications*, *8*(1), 1097, doi:10.1038/s41467-017-01256-2.

Maccaferri, F., E. Rivalta, D. Keir, and V. Acocella (2014), Off-rift volcanism in rift zones determined by crustal unloading, *Nature Geosci*, *7*(4), 297-300, doi:10.1038/ngeo2110.

Menand, T., and S. R. Tait (2002), The propagation of a buoyant liquid-filled fissure from a source under constant pressure: An experimental approach, *Journal of Geophysical Research B: Solid Earth*, *107*(11), doi:10.1029/2001JB000589.

Pinel, V., A. Carrara, F. Maccaferri, E. Rivalta, and F. Corbi (2017), A two-step model for dynamical dike propagation in two dimensions: Application to the July 2001 Etna eruption, *Journal of Geophysical Research: Solid Earth*, *122*(2), 1107-1125, doi:10.1002/2016JB013630.

Sweeney, M. R., and G. A. Valentine (2015), Transport and mixing dynamics from explosions in debris-filled volcanic conduits: Numerical results and implications for maar-diatreme volcanoes, *Earth and Planetary Science Letters*, *425*, 64-76, doi:http://dx.doi.org/10.1016/j.epsl.2015.05.038.

Taisne, B., and S. Tait (2009), Eruption versus intrusion? Arrest of propagation of constant volume, buoyant, liquid-filled cracks in an elastic, brittle host, *J. Geophys. Res.*, *114*(B6), B06202, doi:10.1029/2009jb006297.

Valentine, G. A., and B. van Wyk de Vries (2014), Unconventional maar diatreme and associated intrusions in the soft sediment-hosted Mardoux structure (Gergovie, France), *Bulletin of Volcanology*, *76*(3), 807, doi:10.1007/s00445-014-0807-9.

White, J. D. L., and P. S. Ross (2011), Maar-diatreme volcanoes: A review, *Journal of Volcanology and Geothermal Research*, *201*(1–4), 1-29, doi:http://dx.doi.org/10.1016/j.jvolgeores.2011.01.010.

Woolsey, T. S., M. E. McCallum, and S. A. Schumm (1975), Modeling of diatreme emplacement by fluidization, *Physics and Chemistry of the Earth*, *9*, 29-42, doi:https://doi.org/10.1016/0079-1946(75)90004-X.

REVIEWERS' COMMENTS:

Reviewer #1 (Remarks to the Author):

I thank the authors for taking the previous comments on board and adjusting their article accordingly. They have taken a lot of care to improve the manuscript and to explain their responses to the points raised. The paper is interesting as the results can be applied to volcanic explosions in general, rather than just a feature of diatreme-forming eruptions (which tend to be small and of limited impact), and this is more directly stated in the abstract and introductory paragraphs. Overall, I really like the paper and think it clearly adds to the discussion about stress states, their modulation, and their impact on volcanic plumbing system development and volcanism. This will be an important work and a strong position to lead from in the development of future models.

In my opinion the authors have managed successfully to rephrase their article so that its importance is more clearly stated, but it has the potential to go further. I have a few comments below that I would like addressed (one of which was not fully addressed previously). If this can be done then I would support the publication of this paper in Nature Communications.

Impact of the research
Is there any geophysical evidence that the processes you describe have been recorded during an eruption (i.e. magma intrusion pathways modified after vent formation/during vent filling?). Your Hopi Buttes example in the supplementary materials shows a case study from an ancient system, this is of course required to get geological observations of the plumbing system, but I am wondering if you can try to link with geophysical datasets (e.g. seismicity, ground deformation etc) of recent events. If you can relate more directly to recent events this would strengthen the impact of the paper.
I would like to see some text added on the direct impact that this topic has, for example on affected populations (New Zealand and Mexico are mentioned, what are the affected populations?).

Minor comments
" I would suggest the title of the second section 'stress states during maar-diatreme formation' needs some modification as one might think this is only the excavation stage. Perhaps adding sub-headings (excavation, infilling) might help, but certainly the second paragraph needs a little more explanation at the start that now the infilling stage is considered.
" Figure 2 caption: A and b) should state thick-dashed lines (not solid).
" As I mentioned in my previous review, in Figure S13b) the vertical protrusion coloured in red is not labelled. There does not appear to be a break between the dike and sill and this protrusion. Please include a split in your white 'flow' arrow to include this vertical intrusion and label the occurrence of this hybrid structure.

REVIEWERS' COMMENTS:

Reviewer #1 (Remarks to the Author):

I thank the authors for taking the previous comments on board and adjusting their article accordingly. They have taken a lot of care to improve the manuscript and to explain their responses to the points raised. The paper is interesting as the results can be applied to volcanic explosions in general, rather than just a feature of diatreme-forming eruptions (which tend to be small and of limited impact), and this is more directly stated in the abstract and introductory paragraphs. Overall, I really like the paper and think it clearly adds to the discussion about stress states, their modulation, and their impact on volcanic plumbing system development and volcanism. This will be an important work and a strong position to lead from in the development of future models.

In my opinion the authors have managed successfully to rephrase their article so that its importance is more clearly stated, but it has the potential to go further. I have a few comments below that I would like addressed (one of which was not fully addressed previously). If this can be done then I would support the publication of this paper in Nature Communications.

We would like to thank the reviewer for his great review, which has improved tremendously the quality of our manuscript and brought it to the standard of Nature Communications.

Impact of the research

Is there any geophysical evidence that the processes you describe have been recorded during an eruption (i.e. magma intrusion pathways modified after vent formation/during vent filling?). Your Hopi Buttes example in the supplementary materials shows a case study from an ancient system, this is of course required to get geological observations of the plumbing system, but I am wondering if you can try to link with geophysical datasets (e.g. seismicity, ground deformation etc) of recent events. If you can relate more directly to recent events this would strengthen the impact of the paper.

No phreatomagmatic, diatreme-forming eruptions have been analyzed using modern-day monitoring tools, and thus we depend on our field examples for this eruption style, as well as existing knowledge of vent shifts from other volcanological studies. However, we now provide examples of polygenetic volcanoes with interconnected dike-sill feeder systems, as well as those producing syn-eruptive sill transitions from geodetic observations. We also hope that this model will provide ideas to improve the interpretation of existing or new deformation data that will be recorded at future crater-forming eruptions.

Lines 177-180: "For instance, syn-eruptive sill transitions and shallow magma diversion have been recorded seismically and geodetically at composite volcanoes (e.g., Stromboli; Italy[1]), and sill/laccolith inflation was detected after the onset of explosive volcanism during the 2011 eruption of Cordón Caulle, Chile[2]."

I would like to see some text added on the direct impact that this topic has, for example on affected populations (New Zealand and Mexico are mentioned, what are the affected populations?).

We have updated the manuscript:

Line 33-38: The combination of explosive volcanic activity (e.g., base surges)[3,4,5] and potential vent-site shifts[6,7], which gradually increase the surface region impacted by an eruption, is highly hazardous to densely populated areas situated on volcanic fields (e.g., Mexico City, Mexico, and Auckland, New Zealand). The feedbacks between magma plumbing system development and the location of magma fragmentation must therefore be well-understood to forecast and mitigate volcanic hazards[8,9].

Line 194-199: Our results suggest that while the diversion of magma during hazardous explosive eruptions may interrupt the eruptive sequence it does not necessarily indicate the end of an eruption, and can presage a shift in the vent location[6,7]. Such vent-site shifts should be considered in volcanic hazard mapping and eruptive-response scenarios[8]; detection of shifts and their precursors can be improved through more intensive geophysical and geodetic monitoring[2,10].

Minor comments

" I would suggest the title of the second section 'stress states during maar-diatreme formation' needs some modification as one might think this is only the excavation stage. Perhaps adding sub-headings (excavation, infilling) might help, but certainly the second paragraph needs a little more explanation at the start that now the infilling stage is considered."

We have updated the manuscript.

Line 76: Stress states during diatreme excavation and infilling

Line 93-94: Immediately following a major diatreme-excavation explosion, the diatreme structure is partly re-filled with vertically ejected volcaniclastic material.

Figure 2 caption: A and b) should state thick-dashed lines (not solid).

We have changed the manuscript accordingly.

" As I mentioned in my previous review, in Figure S13b) the vertical protrusion coloured in red is not labelled. There does not appear to be a break between the dike and sill and this protrusion. Please include a split in your white 'flow' arrow to include this vertical intrusion and label the occurrence of this hybrid structure.

Added. We now continue the vertical arrow past the sill diversion, and keep the horizontal arrow to show that this is a hybrid intrusion. The protrusion discussed by the reviewer represents the eroded top to the dike that is feeding the sill in the central part of the image.

1. Casagli N*, et al.* Deformation of Stromboli Volcano (Italy) during the 2007 eruption revealed by radar interferometry, numerical modelling and structural geological field data. *Journal of Volcanology and Geothermal Research* **182**, 182-200 (2009).

2. Castro JM, Cordonnier B, Schipper CI, Tuffen H, Baumann TS, Feisel Y. Rapid laccolith intrusion driven by explosive volcanic eruption. *Nature Communications* **7**, 13585 (2016).

3. Lorenz V. Syn- and posteruptive hazards of maar–diatreme volcanoes. *Journal of Volcanology and Geothermal Research* **159**, 285-312 (2007).

4. Taddeucci J, Sottili G, Palladino DM, Ventura G, Scarlato P. A note on maar eruption energetics: current models and their application. *Bulletin of Volcanology* **72**, 75-83 (2010).

5. Németh K, Cronin SJ, Smith IEM, Agustin Flores J. Amplified hazard of small-volume monogenetic eruptions due to environmental controls, Orakei Basin, Auckland Volcanic Field, New Zealand. *Bulletin of Volcanology* **74**, 2121-2137 (2012).

6. Lefebvre NS, White JDL, Kjarsgaard BA. Spatter-dike reveals subterranean magma diversions: Consequences for small multivent basaltic eruptions. *Geology* **40**, 423-426 (2012).

7. Ort MH, Carrasco-Núñez G. Lateral vent migration during phreatomagmatic and magmatic eruptions at Tecuitlapa Maar, east-central Mexico. *Journal of Volcanology and Geothermal Research* **181**, 67-77 (2009).

8. Lindsay J, Marzocchi W, Jolly G, Constantinescu R, Selva J, Sandri L. Towards real-time eruption forecasting in the Auckland Volcanic Field: application of BET_EF during the New Zealand National Disaster Exercise 'Ruaumoko'. *Bulletin of Volcanology* **72**, 185-204 (2010).

9. Sandri L, Jolly G, Lindsay J, Howe T, Marzocchi W. Combining long- and short-term probabilistic volcanic hazard assessment with cost-benefit analysis to support decision making in a volcanic crisis from the Auckland Volcanic Field, New Zealand. *Bulletin of Volcanology* **74**, 705-723 (2012).

10. Aoki Y, Takeo M, Ohminato T, Nagaoka Y, Nishida K. Magma pathway and its structural controls of Asama Volcano, Japan. *Geological Society, London, Special Publications* **380**, 67-84 (2013).
